# Abstracts of the Fourth Brainstorming Research Assembly for Young Neuroscientists (BraYn), Italy, 20–22 October 2021

**DOI:** 10.3390/neurolint14010010

**Published:** 2022-01-14

**Authors:** Giovanni Ferrara

**Affiliations:** Experimental Neuroscience Lab, IRCCS Ospedale Policlinico San Martino, 16132 Genoa, Italy; Giovanni.Ferrara@hsanmartino.it

**Keywords:** neuroinflammation, neurodegeneration, neuro-oncology, paediatric neuroscience

## Abstract

On behalf of the BraYn Association, we are pleased to present the Abstracts of the Fourth Brainstorming Research Assembly for Young Neuroscientists, which was held from 20–22 October 2021. We congratulate all the presenters on their research work and contribution.

**All abstracts and information are published as submitted by the authors and are not the responsibility of the *Neurology International* journal or the meeting organizer.**

## Whole-Brain Functional Dynamics in Normal Aging during Resting Conditions

Manuela Moretto ^1,^*, Erica Silvestri ^2^, Maurizio Corbetta ^1^ and Alessandra Bertoldo ^2^

^1^ Padova Neuroscience Center, Università degli Studi di Padova, Padova, Italy
^2^ Dipartimento di Ingegneria Dell’informazione, Università Degli Studi di Padova, Padova, Italy
* Correspondence: manuela.moretto@phd.unipd.it

Normal aging is associated with brain structural and functional changes. Employing functional magnetic resonance imaging (fMRI) data acquired in resting state (rs), previous studies applied static functional connectivity (FC) analysis and showed a link between the increase of inter-network connectivity and aging, thus suggesting a reorganization of resting state networks (RSN) in a more integrated topology. However, during the scanning session, the brain transits in and out of different states, and the FC between networks has proven to be time-varying. In this study, we employed a data-driven approach called Hidden Markov Model (HMM) to investigate brain states dynamics in the healthy aging population. We used rs-fMRI data of 88 healthy subjects, equally divided in young and old subjects. Firstly, an independent component analysis was conducted to obtain a whole-brain functional parcellation of the main RSN, and then, the time courses of the RSN were used as input of the HMM. Six brain states were inferred and characterized in terms of FC and mean activity. A graph-based analysis applied on the six FC maps revealed that the age progression leads to a decrease in strength of the default mode network and fronto-parietal network. Moreover, an overall more integrated topology of states occupied by old subjects was observed and in particular between the dorsal attention network and other functional domains. At the single-subject level, we derived the sequence of visited states and the rate of switching between them. We found that two states were mostly occupied by young subjects, whereas three states by old subjects. The transitions between states were not random but followed preferential paths. These results suggest that HMM is able to capture the dynamic transition patterns between brain states and that the aging process has a strong impact in the reorganization of brain functional networks.

## Using the Central Vein Sign and Diffusion MRI to Differentiate Demyelinating from Chronic Vascular Lesions in Multiple Sclerosis

Caterina Lapucci ^1,^*, Silvia Rebella ^2^, Francesc Tazza ^3^, Luca Roccatagliata ^4^, Nicola Mavilio ^4^, Giacomo Boffa ^3^, Elvira Sbragia ^3^, Nicolo’ Bruschi ^3^, Elisabetta Mancuso ^3^, Maria Cellerino ^3^, Simona Schiavi ^3^ and Matilde Inglese ^5^

^1^ HNSR, IRCCS Ospedale Policlinico San Martino, Genova, Italy
^2^ University of Genoa, Genoa, Italy
^3^ DINOGMI, University of Genoa, Genoa, Italy
^4^ Department of Neuroradiology, IRCCS Ospedale Policlinico San Martino, Genoa, Italy
^5^ DINOGMI, IRCCS Ospedale Policlinico San Martino, University of Genoa, Genoa, Italy
* Correspondence: lapuccicate@gmail.com

The impact of vascular risk factors (VRFs) in interpreting the Central Vein Sign (CVS) in Multiple Sclerosis (MS) has been poorly investigated. The aim of the study is to evaluate VRFs impact on the percentage of CVS+/− lesions (%CVS+/−) detected on whole brain and subregions and to investigate whether diffusion MRI metrics are able to differentiate CVS+ from CVS− lesions. A total of 120 MS pts were stratified by age in four groups. 3DEPI-T2*-weighted and multishell diffusion images (ac- quired at 3T) were analysed for the presence of the CVS. A linear regression model was used to predict VRFs impact on the %CVS+ on whole brain and subregions. DTI and NODDI metrics able to differentiate CVS+ from CVS− lesions were identified using ANCOVA. Group 1 (>60 y), 2 (45–60 y), 3 (30–45 y), and 4 (18–30 y) included 30 pts, respectively. The median frequency of CVS+ lesions was 73.5%. The %CVS+ lesions was higher in infratentorial and periventricular areas (*p* = 0.000), while %CVS− lesions was higher in juxtacortical and deep subcortical (ds) white matter (WM) regions (*p* = 0.001 and *p* = 0.002). The %CVS− in dsWM was significantly higher than %CVS+ only in Group1 (*p* = 0.002). The regression model showed that age was predictor of whole-brain %CVS+ (R^2^ = 0.23; *p* = 0.000). In patients with age > 45 y, hypertension (HP) was predictor of %CVS− in dsWM (R^2^ = 0.12; *p* = 0.031). Mean diffusivity (MD) was higher in CVS+ than CVS− lesions (*p* = 0.004). Intracellular volume fraction (ICVF) was lower, and isotropic volume fraction (isoVF) was higher in CVS+ than CVS− lesions (*p* = 0.000 for both). Age and HP showed a relevant impact on the prevalence of CVS− lesions on whole brain and dsWM. The predominance of CVS− lesions in the dsWM, especially in adult-to-elderly MS pts with VRFs, should be considered a “red flag” for concomitant causes of WM damage different from MS. MD, ICVF, and isoVF differentiated CVS+ from CVS− lesions. IsoVF may reveal the presence of more pronounced inflammatory component inside CVS+ lesions.

## Microglial Spatio-Temporal Heterogeneity in a Perinatal Inflammation Mouse Model—Link to Autism-like Phenotypes

Cindy Bokobza ^1,^*, Anne Galland ^1^, David Guenoun ^1^, Alice Jacquens ^1,2^, Valérie Faivre ^1^, Zsolt Csaba ^1^, Leslie Schwendimann ^1^, Sophie Lebon ^1^, Nicolas Heck ^3^, Claire Leconte ^4^, Valérie C. Besson ^4^, Thomas Bourgeois ^1^, Nelina Ramanantsoa ^1^, Boris Matrot ^1^, Jorge Gallego ^1^, Bobbi Fleiss ^1,5^, Juliette Van Steenwinckel ^1,^*^,^^†^ and Pierre Gressens ^1,^*^,†^

^1^ Université de Paris, NeuroDiderot, Inserm, F-75019 Paris, France
^2^ Neuro Intensive Care, Pitié Salpétrière Hospital, F-75013 Paris France
^3^ Neuroscience Paris Seine, Institut de Biologie Paris-Seine, CNRSUMR8246/INSERMU1130, Sorbonne Université, Paris, France
^4^ EA4475—Pharmacologie de la Circulation Cérébrale, Faculté de Pharmacie de Paris, Université de Paris, F-75006 Paris, France
^5^ School of Health and Biomedical Sciences, RMIT University, Bundoora, VIC 3083, Australia
* Correspondence: cindy.bokobza@inserm.fr
† These authors contributed equally to this work.

A general consensus regarding neurodevelopmental disorders, including Autism Spectrum Disorder (ASD), is that they originate from early development defects in brain formation, leading to altered neuronal circuitry responsible for the pathological behaviour. Despite studies on genetic implication in ASDs, a causal relationship between genomic alteration and ASD has been difficult to explain in many cases, suggesting environmental factors might be involved. In fact, preterm birth is often linked to the occurrence of inflammation, and preterm infants have a ten-times higher risk of developing ADS-like symptoms than infants born at term. Moreover, some clinical studies reported ongoing neuroinflammation processes in different brain regions in autistic infants, including frontal cortex, hippocampus, and cerebellum. The major relay of the environmental response in the brain, including inflammatory responses, is microglia cells (MG), the brain-resident macrophages that continuously survey their local environment. Moreover, during development microglia play a critical role during the synaptic pruning to contribute to the formation of the mature cerebral connectivity network. In an inflammatory context, MG are activated and participated to the local release of pro-inflammatory cytokines. Our hypothesis is, therefore, that an exposition to perinatal inflammation impacts on neurodevelopmental disorder symptoms, leading to ASD. Using a mouse model of perinatal inflammation induced by IL1b injection between postnatal day (P)1–5, this project demonstrates that (i) there is region specific inflammation between frontal cortex, hippocampus, and cerebellum determinate; (ii) an impact of microglial activation on the synaptic pruning at P15 and a modulation of connectivity by UltraFast Doppler at P40; and (iii) an impact of the perinatal inflammation on the onset of ASD-like phenotypes at different developmental stages by UltraSonic Vocalization (P2 and P8), Nest Odour preference test (P8), and an adapted three-chamber test (P40). This innovative project has as objective to identify potential diagnosis markers to facilitate an early detection of ASD in premature infants based on inflammatory indicators.

## Electroencephalographic Alterations in SARS-CoV-2-Positive Persons

Paolo Onorati and Ginevra Toma *

Department di Fisiologia e Farmacologia “V. Erspamer,” Università degli studi di Roma La Sapienza, Roma, Italy
* Correspondence: ginevra.toma@uniroma1.it

**Introduction**—The recent severe acute respiratory syndrome coronavirus 2 (SARS-CoV-2, previously known as 2019-nCoV) is a zoonotic virus capable of causing an acute respiratory infectious disease and primarily spreading through the respiratory tract. The current data suggest an incubation period of 1–14 days, in most cases 3–7 days. The virus is highly transmissible human to human and causes severe problems, especially in the elderly and people with underlying chronic diseases. The ACE-2 receptors have been identified as likely infection points for the SARS-CoV-2, and they are broadly expressed in the vascular endothelium, respiratory epithelium, alveolar monocytes, macrophages, neurons, and glial cells. COVID-19 patients typically present with specific, similar symptoms, such as fever, malaise, cough, and neurological signs, such as headache, anosmia, nausea, vomiting, nystagmus, objective vertigo, and convulsions. Here, we used EEG for assess the brain involvement in this viral pathology.

**Methods**—At COVID-Centre 3 of Rome, we recorded 15 EEG in patients with positive at COVID 19 (SARS-CoV-2). We recorded their breathing in ambient air (FiO_2_ 21%), without fever, and five minutes before EEG recording; they also performed an arterial blood gas analysis for testing their effective saturation, partial pressure of carbon dioxide (CO_2_), and oxygen (O_2_), PH, bicarbonate (HCO3−), and haemoglobin level. All patients were in remission of infectious disease and the pneumonia. EEG data were recorded on individuals in a resting state with activation tests (SLI and HPN), through 19 electrodes placed on the scalp, according to the international 10/20 system (electrodes: Fp1, Fp2, F7, F3, Fz, F4, F8, T3, C3, Cz, C4, T4, T5, P3, Pz, P4, T6, O1, and O_2_, linked ears as reference).

**Results**—The EEG of the 15 persons subjected to the study gave asymmetrical results (and therefore is pathological), with a prevalence of signal on the left side of the brain, a condition not in relationship with the respiratory pathology or a poor cerebral blood perfusion, considering the excellent haemodynamic and saturation values of participants. The pattern most represented in this group of patients is characterized by a simultaneous presence of the irritative elements as well as a general slowing of the basic cerebral electrogenesis.

**Conclusions**—The presence of viruses inside the glia can drastically modify brain electrogenesis in the sense of a discontinuity of the basic rhythm. The evidences deriving from these initial observations lead to identify in this virus a pathogenic element that strongly damages the brain structures, capable of causing inflammation, on the one hand, altering the physiology of the basal cerebral electrogenic activity by slowing its functioning and, on the other hand, creating an inflammatory and irritative state that is sometimes clearly visible and sometimes subclinical. Our study will continue with the acquisition of a greater number of data and the possibility of carrying out the follow ups of the persons participating in order to assess the actual involvement of the brain in this viral pathology.

## β3-Adrenergic Receptor Expressing Stromal Cells in Thymus Control Treg Generation and Release of Newly Generated Lymphocytes

Maria Cristina Mariani ^1,^*, Tiziana Vigo ^1^, Consuelo Venturi ^1^, Erika Ricci ^1^, Federico Ivaldi ^2^, Nicole Kerlero de Rosbo ^2^ and Antonio Uccelli ^1^

^1^ Neuroscience Lab., IRCCS San Martino Hospital, Genoa, Italy
^2^ DINOGMI, University of Genoa, Genoa, Italy
* Correspondence: marianimariacristina@gmail.com

The thymus is composed of spatially discrete areas, each of which is characterized by the presence of particular stromal cells, including mesenchymal stem cells (MSC) and T lymphocyte precursors at defined maturation stage. The thymus receives extensive innervation by the sympathetic nervous system (SNS). Norepinephrine (NE) released by the SNS in thymus impacts on α and β2 adrenergic receptors (AR) expressed by thymocytes, controlling selection processes and the specification of maturing lymphocytes into CD8-positive cells. In the bone marrow, NE activates β3AR, which are selectively expressed by MSC, blocking their expression of Cxcl12 and affecting haematopoiesis. In thymus β3AR, expression by stromal cells and effects of its activation on T-cell maturation has never been investigated. Here, we speculated that SNS may promote T-cell generation through NE-mediated activation of β3AR expressed by stromal cells in thymus. To assess our hypothesis, we performed a confocal and FACS analysis of thymic stromal cells in naive mice treated or not with a selective agonist of β3AR. We demonstrate that β3AR is expressed by different subsets of stromal cells in thymus, the majority of which co-expressed the endothelial marker CD31, while a small percentage were positive for the MSC marker, stem cell antigen-1 (Sca1). A confocal analysis of the thymus revealed that β3AR+-expressing cells are present in the medulla and form cell clusters within the external cortex. Activation of B3AR reduced the expression of Cxcl12, promoted the release of newly generated T lymphocytes into circulation and increased the frequency of regulatory T (Treg) cells in the thymus. Overall, our results indicate that the SNS can control the functionality of the thymus through a mechanism that involves β3AR-expressing stromal cells.

## Exercise Protects from Hippocampal Inflammation and Neurodegeneration in Experimental Autoimmune Encephalomyelitis

Livia Guadalupi ^1,^*, Francesca Romana Rizzo ^1^, Krizia Sanna ^1^, Valentina Vanni ^2^, Diego Fresegna ^2^, Francesca De Vito ^3^, Alessandra Musella ^4^, Silvia Caioli ^3^, Sara Balletta ^1^, Silvia Bullitta ^1^, Antonio Bruno ^1^, Ettore Dolcetti ^1^, Mario Stampanoni Bassi ^3^, Fabio Buttari ^3^, Luana Gilio ^3^, Georgia Mandolesi ^4^, Diego Centonze ^1^ and Antonietta Gentile ^2^

^1^ Laboratorio di Immunopatologia Sinaptica, Università Degli Studi di Roma Tor Vergata, Roma, Italy
^2^ Laboratorio di Immunopatologia Sinaptica, IRCSS San Raffaele Pisana, Roma, Italy
^3^ Unità di Neurologia, IRCCS Neuromed, Pozzilli, Italy
^4^ Dipartimento di Scienze Umane e Promozione Della Qualità Della Vita, Università di Roma San Raffaele, Roma, Italy
* Correspondence: livia.guadalupi@gmail.com

Exercise training is increasingly recognized as a valuable strategy to promote wellness in people with multiple sclerosis (MS), a chronic inflammatory neurodegenerative and demyelinating disease. Clinical evidence and data from the animal model of MS, the experimental autoimmune encephalomyelitis (EAE), reveal that exercise can slow down disease progression and pathology. Hippocampal dysfunction represents a pathological feature of MS accounting for cognitive deficits. An inflammation-induced aberrant synaptic plasticity caused by GABAergic transmission reduction, associated with the loss of inhibitory parvalbumin-positive (PV+) interneurons, is proposed to contribute to hippocampal pathology in EAE/MS. Of note, the hippocampus is a brain area highly sensitive to the effects of exercise. Here, we addressed the effects of preventive voluntary running wheel on EAE hippocampal dysfunction, evaluating behavioural, electrophysiological, biochemical, and immunohistochemical outcomes. Our results show that exercise significantly improved clinical disability and corrected cognitive deficits in both presymptomatic and acute-phase EAE, as highlighted by better performance of exercise-EAE mice at novel object-recognition task and nest-building test, respectively. Exercise was shown to correct the EAE-induced aberrant hippocampal plasticity measured by field potential recording by counteracting the PV+ interneuron degeneration and by attenuating inflammation. On one side, voluntary running wheel exerted a relevant neuroprotective action. On the other side, exercise reduced hippocampal microgliosis and the expression of tumour necrosis factor in microglia and, to a lesser extent, the hippocampal level of interleukin 1beta, previously shown to contribute to the aberrant synaptic plasticity in the EAE hippocampus. Overall, these data provide evidence that physical exercise improves cognitive function and prevents synaptic and neuronal damage that typically affect EAE/MS hippocampus.

## Developing Silk Scaffold-Based Platform to Generate Functional and Reproducible Human Bioengineered Forebrain Organoids

Edoardo Sozzi *, Janko Kajtez, Andreas Bruzelius, Petter Storm, Daniella Ottosson, Malin Parmar and Alessandro Fiorenzano

Department of Experimental Medical Science, Lund University, Box 117, 221 00 Lund, Sweden
* Correspondence: edoardo.sozzi@med.lu.se

Three-dimensional (3D) human brain organoids have rapidly become a widely used system to study brain development in a dish. Cultured over long periods of time, brain organoids provide a unique opportunity to model mature neuronal features, including cytoarchitecture and cell-cell interactions reminiscent of human brain complexity. However, conventional 3D methodology is hampered by high variability in terms of morphology, size, and cellular composition and the presence of immature differentiation in the inner core. Therefore, we established a novel technological approach, using recombinant silk protein to create a bioengineered scaffold that arranges hPSCs in an organ-like configuration while maintaining their self-organizing property. We showed that silk scaffold sustained the homogeneous differentiation into mature neurons throughout all compartments of the organoid, avoiding spontaneous differentiation of cells towards meso-endodermal fate as occasionally observed in conventionally generated organoids. Whole-cell patch clamp recordings together with calcium imaging confirmed the presence of an intricate neuronal network of functionally active neurons. Furthermore, by using optical oxygen sensors that can be easily integrated into 3D cultures, we measured the oxygen gradients in silk bioengineered and conventional organoids. Our findings showed the remarkable property of silk scaffolds to form porous microarchitectures facilitating the delivery of oxygen, nutrients, and extrinsic patterning cues, thus creating more favourable growth and differentiation conditions.

## The Intranasal Administration of Cholesterol as a Possible Therapeutic Strategy in Huntington’s Disease

Monica Favagrossa ^1,^*, Alice Passoni ^1^, Laura Colombo ^1^, Renzo Bagnati ^1^, Giulia Birolini ^2^, Marta Valenza ^2^, Elena Cattaneo ^2^ and Mario Salmona ^1^

^1^ Istituto di Ricerche Farmacologiche Mario Negri IRCCS, Via Mario Negri 2, 20156 Milan, Italy
^2^ Department of Biosciences, University of Milan, Via G. Celoria 26, 20133, Milan, Italy
* Correspondence: monica.favagrossa@marionegri.it

Huntington’s disease (HD) is a dominant neurodegenerative disorder characterized by neuronal dysfunction and cell loss. One of the affected pathways implicates brain cholesterol (chol) metabolism, and exogenous chol administration to HD mice ameliorates their phenotype, indicating chol as a good candidate for HD treatment. Considering that the strategies used are invasive and not easily transferable to the patients, we decided to combine the safety of the intranasal (IN) technique with the administration of liposome-loaded chol, whose formulation has already been used for commercial drugs.

WT and R6/2 mice were treated with a single dose of liposome-loaded cholesterol-D6 (chol-D6) (200 μg chol-D6/dose) during the acute trial. The LC-MS analysis confirmed the delivery of chol-D6 to the whole brain thought IN route independently from genotype by reaching a stable concentration until 10 days after IN treatment (0.4 ng/mg). Chol-D6 rose in the first 24 h in the peripheral tissues and declined ten days after IN treatment. At 42 days after IN treatment, chol-D6 level in the striatum of R6/2 mice was statistically reduced than WT mice, suggesting that exogenous chol supplied the lack of chol in R6/2 mice.

During the chronic trial, a group of 5-week-old R6/2 mice received 7 IN doses of chol-D6 (200 μg chol-D6/IN) once a week, and at the same time, two control groups (WT and R6/2 mice) received PBS. The LC-MS analysis confirmed the chol-D6 accumulation after IN repeated treatments in the brain areas (about 3.5 ng/mg). R6/2 mice treated with liposomes rescued cognitive decline, while their strength were statistically improved compared to R6/2 treated with PBS.

This result highlighted the accumulation of chol-D6 in the whole brain, while its excess was eliminated from the peripheral tissues. Moreover, repeated IN treatments rescued cognitive functions and counteracted the muscular strength defect.

## Investigating a New Therapeutic Role of the GHRH Agonist MR409 in an Experimental Model of Spinal Muscular Atrophy

Anna Caretto ^1,^*, Iacopo Gesmundo ^2^, Roberta Schellino ^1^, Andrew Viktor Schally ^3^, Riccarda Granata ^2^, Marina Boido ^1^ and Alessandro Vercelli ^1^
^1^ Department of Neurosciences “Rita Levi Montalcini”, Neuroscience Institute Cavalieri Ottolenghi, University of Turin, Orbassano, Italy

^2^ Department of Medical Sciences, Division of Endocrinology, Diabetes, and Metabolism, University of Turin, Turin, Italy
^3^ Department of Medicine, Division of Endocrinology, Miller School of Medicine, University of Miami, Miami, FL, USA
* Correspondence: anna.caretto@unito.it

Spinal muscular atrophy (SMA) is a neurodegenerative genetic disease characterized by a progressive atrophy of skeletal muscles. It is caused by a reduction of survival motor neuron (SMN) protein levels that leads to lower motor neuron (MN) loss. Nowadays, the investigation of *SMN*-independent treatments is spreading ever more to bypass the limitations of the already available therapies, such as difficult administration, several adverse effects, and high costs. Here, we evaluated the role of MR409, a growth hormone-releasing hormone (GHRH) agonist that has shown to be able in preventing apoptosis and proteolysis in an in-vitro model of muscle atrophy. Therefore, from postnatal day 2 (P2) to P12, we daily administered vehicle or MR409 (1 mg/Kg and 2 mg/Kg) to SMNdelta7 mice, a model of SMA type II. We observed a progressive gain of weight, especially with the highest dose, and a significant improvement of motor performances in terms of reflexes, strength, and resistance. According to these positive outcomes, histological analysis on quadriceps and gastrocnemius revealed a significant increase in the size of the muscular fibres and moreover a higher rate of neuromuscular junction maturation with an enhanced monoinnervation and a reduced denervation of the endplates. Finally, at the molecular level, we observed an increased expression of several myosin heavy chain isoforms (MYH1, MYH2, MYH7, and MYH8) and of markers of myogenesis and muscular damage repairing (Myogenin and MyoD1) as well as a significant downregulation of apoptosis markers correlated with muscular atrophy (MuRF1 and Atrogin-1). Finally, the highest dose of MR409 seemed to be able in reducing MN loss in lumbar spinal cord and in decreasing astrogliosis rate with a downregulation of proinflammatory cytokine (TNFα, IL-1b and IL-6) release in the same district. Thus, our results suggest MR409 as a new promising therapeutic approach for SMA treatment perhaps in combination with *SMN*-dependent therapies.

## Biallelic Variants in Spart Cause a Severe Mitochondrial Dysfunction Rescued by CoQ10 Complementation

Chiara Diquigiovanni ^1,^*, Antje Kampmeier ^2^, Christian Bergamini ^3^, Nicola Rizzardi ^3^, Alma Kuechler ^2^, Marco Seri ^1^ and Elena Bonora ^1^

^1^ Department Medical and Surgical Sciences (DIMEC), University of Bologna, Bologna, Italy
^2^ Institut für Humangenetik, Universität Duisburg-Essen, Essen, Germany
^3^ Department of Pharmacy and Biotechnology (FABIT), University of Bologna, Bologna, Italy
* Correspondence: chiara.diquigiovanni@unibo.it

Troyer syndrome is an autosomal recessive form of spastic paraplegia resulting in lower extremity spasticity and weakness, short stature and cognitive defects due to loss-of-function mutations in *SPART*. *SPART* encodes for Spartin, a multifunctional protein interacting with microtubules and mitochondria. We previously observed that mutant Spartin caused a mitochondrial dysfunction characterized by Complex I impairment. Performing whole-exome sequencing in a six-year-old boy with short stature, muscle weakness, and developmental delay, we identified two novel compound heterozygous missense variants in *SPART* (both of unknown significance, class 3), one maternally and one paternally inherited. Immunofluorescence staining in control and patient’s fibroblasts revealed a marked nuclear localization of Spartin in the mutant cells, whereas in controls, it was evenly distributed in the cells. In-vitro analysis on the patient’s fibro-blasts showed an altered mitochondrial network, decreased activity of the oxidative phosphorylation system and ATP levels, increased mitochondrial reactive oxygen species production, increased mitochondrial membrane potential, and altered Ca^2+^ levels vs. control fibroblasts. Interestingly, re-expression of *SPART* restored both the ATP/ADP ratio and intracellular Ca^2+^ levels as in controls, providing the evidence that these observed defects were specifically caused by mutated Spartin. Moreover, we found a decreased of coenzyme Q10 (CoQ10) compared to control fibroblasts along with the decrease, in terms of protein expression, of COQ7 and COQ9 (two enzymes involved in the formation of Q10). Supplementing the medium of the patient’s fibroblasts with a membrane permeable CoQ10 formulation, we observed a significant recovery in ATP synthesis and cell growth compared to untreated patient’s and control fibroblasts. These data suggest that CoQ10 supplementation may represent an interesting therapeutic approach for in-vivo treatment.

## Expression of Serum miR-223-3p and miR-7-1-5p in Parkinson’s Disease Patients

Lorenzo Agostino Citterio ^1,^*, Roberta Mancuso ^1^, Simone Agostini ^1^, Mario Meloni ^1^ and Mario Clerici ^2^

^1^ IRCCS Fondazione Don Carlo Gnocchi, Laboratorio di Medicina Molecolare e Biotecnologie, Milano, Italy
^2^ Department of Pathophysiology and Transplantation, Università di Milano, Milano, Italy
* Correspondence: lcitterio@dongnocchi.it

Parkinson’s disease (PD) is the most common movement disorder, affecting about 6 million individuals worldwide. The aetiology of PD is still poorly understood, mainly due to its multifactorial nature where genetic and environmental interaction seems to play a fundamental role. Recently, microRNAs (miRNAs) have been shown to be important biological molecules involved in diverse processes to maintain normal cellular functions. Over the past decade, many studies have reported dysregulation of miRNA expressions in PD. In particular, miRNAs extracted in circulatory fluids could represent potential biomarkers for the evaluation of the pathology. We analysed the expression levels of miR-7-1-5p and miR-223-3p, two miRNAs involved in α-Synuclein pathway, extracted from samples of serum collected from a population of 82 subjects, including 41 PD patients and 41 healthy controls (HC), through the use of droplet digital PCR (ddPCR) in order to compare their expression level between the two enrolled groups. We also extracted miR-7-1-5p from serum exosomes, small extracellular vesicles able to cross the blood-brain barrier and with the function of facilitating intracellular communication. Serum miR-7.1.5p was significantly more expressed in PD (32.25; 5.82–71.63 copies/ng) compared to HC (0.00; 0.00–25.10 copies/ng; *p* = 0.0006), while no differences have been found in exosomes. In the same way, the expression of serum miR-223-3p was significantly increased in the PD group (4476.19; 1981.93–8754.85 copies/ng) compared to HC (937.50; 145.19–6605.05 copies/ng; *p* = 0.0007). An interesting correlation was also found between the expression level of serum miR-223 in PD patients and the levodopa equivalent daily dose, or LEDD (mg/die; *p* = 0.0061). L-DOPA is the precursor of dopamine neurotransmitters and, to date, remains the most effective treatment for PD patients. Based on the obtained results, we confirm the usefulness of miRNAs as potential biomarkers to investigate the aetiology of PD.

## Inhibiting Microcephaly Genes as Alternative to Microtubule Targeting Agents to Treat Brain Tumours

Gianmarco Pallavicini ^1,^*, Giorgia Iegiani ^1^, Marta Gai ^2^, Valeria Bitonto ^2^, Roberta Parolisi ^1^ and Ferdinando Di Cunto ^1^

^1^ Department di Neuroscienze “Rita Levi Montalcini”, Neuroscience Institute Cavalieri Ottolenghi, Università di Torino, Torino, Italy
^2^ Molecular Biotechnology Center, Department di Biotecnologie e Scienze per la Salute, Università di Torino, Torino, Italy
* Correspondence: gianmarco.pallavicini@unito.it

Medulloblastoma (MB) and gliomas are the most frequent high-grade brain tumors (HGBT) in children and adulthood, respectively. The general treatment for these tumors consists in surgery, followed by radiotherapy and chemotherapy. Despite the improvement in patient survival, these therapies are only partially effective, and many patients still die. In the last decades, microtubules have emerged as interesting molecular targets for HGBT, as various microtubule-targeting agents have been developed and tested pre-clinically and clinically with encouraging results. Nevertheless, these treatments produce relevant side effects since they target microtubules in normal as well as in cancerous cells. A possible strategy to overcome this toxicity could be to target proteins that control microtubule dynamics but are required specifically by HGBT cells. The genes mutated in primary hereditary microcephaly (MCPH) are ubiquitously expressed in proliferating cells but under normal conditions are selectively required during brain development in neural progenitors. There is evidence that MB and glioma cells share molecular profiles with progenitors of cerebellar granules and of cortical radial glia cells, in which MCPH gene functions are fundamental. Moreover, several studies indicate that MCPH genes are required for HGBT expansion. Among the 25 known MCPH genes, we focused on CENPE and CITK, which have been found to control microtubule stability during cell division and genome stability. Inhibition of this genes lead to cell cycle block, apoptosis, and proliferation arrest in in-vitro and in-vivo models of HGBT. Our data suggest these genes are promising and specific candidates as HGBT targets.

## Microglia-Derived Small Extracellular Vesicles Reduce Glioma Growth by Modifying Tumour Cell Metabolism and Enhancing Glutamate Clearance through miR-124

Carmela Serpe ^1,^*, Lucia Monaco ^1^, Michela Relucenti ^2^, Ludovica Iovino ^3^, Pietro Familiari ^4^, Ferdinando Scavizzi ^5^, Marcello Raspa ^5^, Giuseppe Familiari ^2^, Laura Civiero ^3^, Igea D’Agnano ^6^, Cristina Limatola ^1^ and Myriam Catalano ^1^

^1^ Department of Physiology and Pharmacology, University of Rome Sapienza, Rome, Italy
^2^ Department of Anatomical, Histological, Forensic Medicine and Orthopedics Sciences, University of Rome Sapienza, Rome, Italy
^3^ Department of Biology, University of Padova, Padova, Italy
^4^ Department of Human Neurosciences, University of Rome Sapienza, Rome, Italy
^5^ CNR, Institute of Biochemistry and Cell Biology, Monterotondo, Italy
^6^ CNR, Institute of Biomedical Technologies, Segrate, Italy
* Correspondence: carmela.serpe@uniroma1.it

Glioblastoma (GBM) is one of the most common and malignant kinds of brain cancer. An altered intercellular communication constitutes a base for the onset and the development of the disease. Extracellular vesicles (EVs) are active players in the brain homeostasis, contributing to the continuous exchange of information among neurons, glial cells, and brain immune cells, namely microglia. The major non-neoplastic cell population in GBM microenvironment is represented by tumor-associated macrophages/microglia (TAMs), which can constitute up to 40% of the tumor mass. There are two subtypes of EVs: the medium/large EVs (m/lEVs) and small EVs (sEVs). sEVs released by microglia play an important role in brain patrolling both in physiological and pathological processes. In this work, we analysed the effects of microglia-derived sEVs in GBM by using in-vitro and in-vivo models (murine glioma cells and injection of tumor cells in C57BL6/N mice). Our findings indicated that sEVs carry messages to cancer cells that modify glioma cell metabolism, reducing lactate, nitric oxide (NO), and glutamate (Glu) release, which are all molecules important in supporting tumor growth. Particularly, sEVs affect Glu homeostasis, increasing the expression of Glu transporter Glt-1 on astrocytes. We demonstrated that this effect is mediated by miR-124 contained in microglia-released sEVs. Furthermore, the in-vivo benefit of microglia-derived sEVs results in a significantly reduced tumor mass and an increased survival of glioma-bearing mice, depending on miR-124.

## Tackling Creatine Transporter Deficiency: New Insight into Cell-Specific Vulnerability and Development of a Gene Therapy Approach

Elsa Ghirardini ^1,^*, Francesco Calugi ^2^, Giulia Sagona ^2^, Federica Di Vetta ^3^, Martina Palma ^2^, Roberta Battini ^4^, Giovanni Cioni ^5^, Tommaso Pizzorusso ^2^ and Laura Baroncelli ^1^

^1^ National Research Council (CNR), Institute of Neuroscience, Pisa, Italy
^2^ Department of Neuroscience, Psychology, University of Florence, Drug Research and Child Health (NEUROFARBA), Florence, Italy
^3^ Department of Biology, University of Pisa, Pisa, Italy
^4^ Department of Clinical and Experimantal Medicine, University of Pisa, Pisa, Italy
^5^ Department of Developmental Neuroscience, IRCCS Stella Maris Foundation, Pisa, Italy
* Correspondence: elsa.ghirardini@in.cnr.it

Creatine Transporter Deficiency (CTD) is an X-linked neurodevelopmental disorder caused by mutations in the Creatine Transporter (CrT) gene, presenting with cerebral creatine depletion, intellectual disability, behavioural problems, and epilepsy. Currently, there is no cure for CTD, and the pathogenic mechanisms of the disease remain elusive, hampering the identification of good therapeutic targets. Achieving a better understanding of the bases of CTD and searching for therapies are therefore challenges that need to be addressed in parallel. We generated a mouse model, which faithfully recapitulates the symptoms observed in patients. Based on this tool, we studied how creatine depletion affects the different cell populations of the brain. By combining single-cell RNA sequencing, electrophysiological techniques, and behavioural studies, we found that creatine depletion alters gene expression in specific cell types, with a major impact on parvalbumin inhibitory neurons, causing structural and functional alteration in these cells. Creatine depletion in parvalbumin neurons is sufficient to cause cognitive impairment and increased susceptibility to epilepsy, indicating a fundamental role for these cells in the pathogenesis of CTD. We also evaluated gene therapy as a possible treatment. We used Adeno-Associated Viral vectors to deliver a functional CrT gene (AAV/CrT) to newborn CTD mice. AAV/CrT administration resulted in the expression of transgenic CrT, increasing brain creatine levels and improving cognitive performance. However, toxicity was observed with high titres of the vector. We are currently optimising the vector dosage and design to obtain a widespread, physiological expression of CrT reducing the toxicity caused by creatine overload.

## Environmental Enrichment Modifies Gut Microbiome and Metabolome Enhancing Memory and Neurogenesis through Short-Chain Fatty Acids

Francesco Marrocco ^1,^*, Mary Delli Carpini ^1^, Stefano Garofalo ^1^, Ottavia Giampaoli ^2^, Eleonora De Felice ^1^, Maria Amalia Di Castro ^1^, Laura Maggi ^1^, Ferdinando Scavizzi ^3^, Marcello Raspa ^3^, Federico Marini ^4^, Alberta Tommasini ^4^, Roberta Nicolosi ^2^, Carolina Cason ^5^, Flavia Trettel ^1^, Alfredo Miccheli ^6^, Valerio Iebba ^7^, Giuseppina D’Alessandro ^8^ and Cristina Limatola ^9^

^1^ Department of Physiology and Pharmacology, Sapienza University, Rome, Italy
^2^ Department of Chemistry, Sapienza University, Rome, Italy
^3^ EMMA-CNR, EMMA-CNR, Monterotondo, Italy
^4^ NMR-Based Metabolomics Laboratory (NMLab), Sapienza University, Rome, Italy
^5^ Department of Medical Sciences, University of Trieste, Trieste, Italy
^6^ Department of Enviromental Biology, Sapienza University, Rome, Italy
^7^ Institute of Maternal and Child Health-IRCCS “Burlo Garofalo,” SSA of Advanced Microbiology Diagnosis and Translational Research, Trieste, Italy
^8^ IRCCS-Neuromed, IRCCS-Neuromed, Pozzilli, Italy
^9^ Department of Physiology and Pharmacology, Sapienza University Affilieted to Istituto Pasteur Italia, Rome, Italy
* Correspondence: francesco.marrocco@uniroma1.it

Gut microorganisms and their products thoroughly affect both host behaviour and brain development and function. Since improvement of brain plasticity and cognition have been demonstrated with enriched housing condition with prolonged motor, sensorial, and social stimuli, we hypothesised that gut microbiota and metabolome could be modulated by environmental enrichment, providing part of the missing link among environmental signals and brain effects. Metagenomic and metabolomic analyses of mice housed in standard or enriched environment highlight environment-specific microbial community and metabolic profiles. We observed that mice housed in an enriched environment showed a reduction of gut microbial richness and diversity indexes and were characterized by a metabolomic fingerprint with the increase of two short-chain fatty acids (SCFA) formate and acetate and the decrease of bile salts. Moreover, we demonstrated that mice treated with a mixture of formate and acetate improved hippocampal neurogenesis, neurotrophins expression, and cognitive behaviour, recapitulating some effect of environmental enrichment. These data showed us that SCFA could be molecular effectors of enriched environment in the brain.

## Hippocampal Estrogenic Signalling Mediates Sex Differences in Retroactive Interference

Marco Rinaudo ^1,^*, Fabiola Paciello ^2^, Francesca Natale ^2^, Francesco La Greca ^1^, Domenica Donatella Li Puma ^2^, Salvatore Fusco ^2^ and Claudio Grassi ^2^

^1^ Department di Neuroscienze, Università Cattolica del Sacro Cuore, Roma, Italy
^2^ Department di Neuroscienze, Università Cattolica del Sacro Cuore—Fondazione Policlinico Universitario “A. Gemelli” IRCSS, Roma, Italy
* Correspondence: marco.rinaudo@unicatt.it

Memory loss is the distinctive trait of different neurodegenerative diseases. However, memory removal is a physiological function, and little is known about its molecular and cellular underpinnings. One mechanism for removing information stored in the brain is retroactive interference, a phenomenon in which newly acquired information overwrites or interferes with the retrieval of already stored information. We have observed that, in a different version of the novel object recognition test in which a new couple of objects unrelated to the training couple is experienced prior to the test phase, adult male C57bl/6 mice suffer from retroactive interference and are unable to discriminate the novel object from the old object. On the other hand, age-matched C57bl/6 female mice show resistance to the same interference protocol. Modulation of estrogenic signaling within the dorsal hippocampus during the interference paradigm renders female mice susceptible to interference, suggesting estrogen involvement. Western blot analysis revealed a higher level of activatory phosphorylation of ERK1/2 at Thr202/Tyr204 in the hippocampus of female mice compared to male mice in response to the interference protocol. Analysis of *c-fos* expression within the dorsal hippocampus showed higher activation of the dentate gyrus (DG) in female mice. Finally, injection of an ERK1/2 inhibitor into the dorsal hippocampus of female mice prior to the interference procedure renders females susceptible to the interference-mediated memory loss. Collectively, our data suggest that hippocampal estrogenic signaling may contribute, through ERK1/2 and DG activation, to a pattern separation mechanism that reduces object-related retroactive interference in female mice.

## Incoming and Outgoing Information Flows Relate with Node Functional Strength Both in Human and Mouse Resting State fMRI

Giorgia Baron ^1,^*, Danilo Benozzo ^1^, Elvina Gindullina ^1^, Ludovico Coletta ^2^, Mattia Zorzi ^1^, Alessandro Gozzi ^2^, Maurizio Corbetta ^3^, Alessandro Chiuso ^1^ and Alessandra Bertoldo ^1^

^1^ Department of Information Engineering, University of Padova, Padova, Italy
^2^ Center for Neuroscienceand Cognitive Systems, Istituto Italiano di Tecnologia, UniTn, Rovereto, Italy
^3^ Departmentof Neuroscience, University of Padova, Padova, Italy
* Correspondence: giorgia.baron.2@phd.unipd.it

Brain network analysis with resting-state functional Magnetic Resonance Imaging (fMRI) data commonly uses a network description based on Functional Connectivity (FC), i.e., the statistical dependence between brain regions. This type of connectivity metrics lacks in measuring directed interactions and is biased by the presence of spurious interactions. A much richer description can be obtained through Effective Connectivity (EC). Indeed, EC allows to account for the direction of propagating information, which is interpreted in terms of causal interaction among brain areas. Moreover, Dynamic Causal Modelling (DCM), which is considered the state-of-the-art method to infer EC, provides a biophysical model of the fMRI signal by decomposing it into the underlying neuronal signal and the hemodynamic effect. However, little is known about how FC-and EC-based whole-brain networks relate with each other. In this work, we employed both human and mouse data to apply a recently proposed sparse version of DCM developed for resting-state fMRI, focusing on connectivity network properties at the single-node level. Firstly, particularly in humans, we observed that most EC links are short-range with the exception of the homologous inter-hemispheric interactions. Then, we found that the incoming information of each node positively correlates with the mean node FC strength, while a negative correlation was observed between the incoming and outgoing EC information, meaning that, on average, strong receivers are weak senders and vice versa. On the contrary, FC seems not to relate to outgoing EC. Specifically for humans, cerebellum consistently shows a negative FC correlation with cortical nodes and behaves as an inhibitory outgoing EC hub.

## An Investigation of the Microstructural Connectivity Alterations in MS

Sara Bosticardo ^1,^*, Simona Schiavi ^1^, Sabine Schaedelin ^2^, Po-Jui Lu ^3^, Muhamed Barakovic ^3^, Matthias Weigel ^3^, Ludwig Kappos ^3^, Jens Kuhle ^2^, Alessandro Daducci ^1^ and Cristina Granziera ^2^

^1^ Department of Computer Science, University of Verona, Verona, Italy
^2^ Neurology/Departments of Medicine, University Hospital Basel, University of Basel, Basel, Switzerland
^3^ Department of Biomedical Engineering, Translational Imaging in Neurology (ThINk)/University Hospital Basel, University of Basel, Basel, Switzerland
* Correspondence: sara.bosticardo@univr.it

The map of brain structural connections can be modeled as a graph where nodes correspond to gray matter (GM) regions and edges to the structural connections between them. This formalism allows to extract network metrics to capture pathology-related alterations. In general, connection strength is computed by counting the number of streamlines (NOS) connecting pairs of GM regions. However, recent works have highlighted that this method is not quantitative. In this study, we weighted the connections using diffusion-based microstructural maps to investigate the structural changes in multiple sclerosis (MS) patients’ networks as well as the assessment of the correlations between these changes and clinical disability. We performed the analyses in a group of 66 MS patients (39F, 43.9 ± 14.5 y) and 64 healthy controls (38F, 36.9 ± 12.8 y). The networks were built using deterministic-like tractography; the GM was segmented in 85 regions using T1-weighted images, and the connections strength was computed by averaging along the streamlines paths the value of the microstructural maps. From each connectome, we extracted five global metrics: density (ratio between actual and possible connections); efficiency (inverse characteristic path length); modularity (network segregation); clustering coefficient (degree on which nodes tend to cluster together); and mean strength (average of edge weights connected to a node). We employed a robust linear model using age, sex, and density as covariates. The patients’ connectomes weighted with intra-cellular maps showed a significant reduction in global efficiency, clustering coefficient, and mean strength as well as increased modularity w.r.t. controls (all the *p*-values are below 0.03). Moreover, the increased modularity of patient networks was related to the worsening of motor disabilities (*p*-values < 0.03). Network properties assessed with NOS were neither sensitive to MS pathology nor correlated with clinical measures of disability in MS patients.

## Dependency of Localization Error and Spatial Spread on the Regularization Parameter in the EEG Source Reconstruction Problem

Ilaria Mazzonetto ^1,^*, Stefano Bovo ^2^, Dante Mantini ^3^ and Alessandra Bertoldo ^1^

^1^ Department of Information Engineering, University of Padova, Padova, Italy
^2^ Padova Neuroscience Center, University of Padova, Padova, Italy
^3^ KU Leuven, Movement Control & Neuroplasticity Research Group, Leuven, Belgium
* Correspondence: ilaria.mazzonetto@unipd.it

High-density electroencephalography (EEG) combined with source reconstruction techniques has nowadays become a powerful brain imaging tool. Accuracy of EEG source reconstruction depends on several factors, such as the degree of approximation considered to build the head model and the inverse solution method adopted. Since the inverse problem is ill-posed, a regularization procedure is essential. To the best of our knowledge, no study has investigated the accuracy of the source reconstruction depending on the choice of the regularization parameter when solving the inverse problem. To tackle this issue, we used simulated EEG data with a signal to noise ratio equal to 5, 10, and 15. Firstly, we built a realistic head model based on the segmentation of a structural image considering 256 channels and 40,000 sources homogeneously distributed in the gray matter cortex. Combining information from the head model, channel positions, and dipole locations, we computed the leadfield matrix using the simbio Finite Element Method. Finally, the simulated EEG potentials were obtained by projecting each source onto the scalp sensors using the leadfield matrix and adding Gaussian white noise. For each source, the inverse problem was solved using the Weighted Minimum Norm Estimation method with 30 different regularization parameters (λ) logarithmically spaced between 10−5 and 101. Performances of source reconstructions were quantified by means of the localization error and spatial spread. Our analyses revealed the following: (i) with greater λ, sources are localized with higher precision; (ii) very low and very high levels of regularization yielded more widely distributed solutions; (iii) with both metrics, noisier data require more regularization to achieve the same performance as for cleaner data. The choice of the regularization parameter should therefore be made considering the amount of noise affecting the data.

## Nutritional Overload Worsens EAE Severity by Promoting Synaptic Damage and Neuroinflammation

Sara Balletta ^1,^*, Alessandra Musella ^2^, Silvia Caioli ^3^, Diego Fresegna ^4^, Francesca De Vito ^3^, Valentina Vanni ^4^, Livia Guadalupi ^1^, Francesca Romana Rizzo ^1^, Krizia Sanna ^1^, Antonietta Gentile ^4^, Giuseppe Matarese ^5^, Diego Centonze ^1^ and Georgia Mandolesi ^4^

^1^ Medicina dei Sistemi, Università degli Studi Tor Vergata, Roma, Italy
^2^ Department of Human Sciences and Quality of Life Promotion, University of Rome San Raffaele, Roma, Italy
^3^ IRCCS Istituto Neurologico Mediterraneo, Nuromed, Pozzilli, Italy
^4^ Synaptic Immunopathology Lab, IRCCSSan Raffaele Pisana, Roma, Italy
^5^ Laboratorio di Immunologia, Istitutoperl’Endocrinologia e l’Oncologia Sperimentale—Consiglio Nazionale delle Ricerche Napoli, Napoli, Italy
* Correspondence: balletta.sara@gmail.com

Multiple sclerosis (MS) is the main neurodegenerative autoimmune disease of the central nervous system in young adults. Growing evidence indicates that chronic inflammation promoted by obesity contributes to MS susceptibility and disease severity although the reason for these phenomena is still not completely understood. Recent studies suggest that the “metabolic pressure” induced by nutritional overload could set the basis for an exaggerated immuno-inflammatory response to self, leading to chronic inflammation/autoimmunity in subjects with autoimmunity risk factors. In MS and in its mouse model, experimental autoimmune encephalomyelitis (EAE) inflammatory molecules and downstream mechanisms cause “synaptopathy,” a reversible synaptic dysfunction that later on can cause excitotoxic damage and neuronal death. The aim of this study was to identify the relationship of nutritional overload with neuroinflammation and synaptic damage in EAE in order to understand the influence of obesity on the pathological mechanisms that control the disease course. We explored the impact of a high-fat diet (HFD) compared to a standard diet (SD) in EAE and control mice (*n* = 25 for each experimental group) by monitoring clinical score and by performing behavioural, electrophysiological, and molecular experiments. Our results indicate that HFD caused significant increase of both excitatory transmission and inflammation within the striatum of control mice. As expected, HFDobesity prompted a worsen EAE clinical deficits by increasing clinical score and weight loss dependent on EAE induction. Interestingly, during the acute phase of the disease, the HFD exacerbated the EAE striatal synaptopathy, strongly increasing the duration and the frequency of glutamatergic currents. In parallel, the striatal neuroinflammatory status of EAE mice fed with HFD was significantly enhanced compared to EAE mice fed with SD. Overall, we demonstrated that high-fat diet strongly contributes to the pathogenesis of EAE by altering glutamate signaling and neuroinflammation, the potentially reversible mechanisms that control MS severity.

## Immunometabolic Reprogramming by Tetramerization of Pyruvate Kinase M2 Reduces Dendritic Cell Activation

Marta Bottero ^1,^*, Fabrizio Loiacono ^1^, Stefano Angiari ^2^, Nicole Kerlero de Rosbo ^3^, Antonio Uccelli ^1,3^ and Giovanni Ferrara ^1^

^1^ IRCCS Ospedale Policlinico San Martino, L. go R. Benzi, 10, 16132 Genoa, Italy
^2^ Otto Loewi Research Center, Division of Immunology and Pathophysiology, Medical University of Graz, Graz, Austria
^3^ Department of Neurology, Rehabilitation, Ophthalmology, Genetics, Maternal and Child Health, University of Genoa, L.go P. Daneo, 3, 16132 Genoa, Italy
* Correspondence: marta.bottero@hsanmartino.it

The last step of the metabolic pathway that converts glucose into pyruvic acid (glycolysis) is regulated by the enzyme pyruvate kinase (PK). In mammals, four isoforms of PK have been identified, and recent studies highlighted the peculiar activity of the isoform PK muscle 2 (PKM2). The enzymatic activity of PKM2 is dependent on its oligomerization state, including active tetramer, less active dimer, and inactive monomer. PKM2 displays both metabolic and non-metabolic functions; on one hand, in the cytoplasm, PKM2 catalyzes the production of pyruvate, and on the other hand, PKM2 in the nucleus may regulate the transcription of several genes directly or by affecting the functionality of other transcription factors. Dendritic cells (DCs) play a crucial role in immune system activation and during inflammation, immature DCs become activated expressing molecules required for DC migration, antigen presentation, and T-cell activation. Upon activation per se and in experimental autoimmune encephalomyelitis (EAE), glycolysis is increased in DCs, supporting a metabolic switch from oxidative phosphorylation (resting DCs) to glucose intake. These observations indicate that metabolic changes occurring in immune cells have a crucial role in their effector responses; accordingly, targeting immune-metabolism is considered an anti-inflammatory strategy. Therefore, the aim of this project is to reduce the activation of DCs using a PKM2 activator, leading to a reduced activation of T cells and, consequently, of the encephalitogenic response in EAE. To explore the role of PKM2 in DC activation, we used an activator, TEPP-46, which stabilizes PKM2 in its tetrameric form. To evaluate the role of PKM2 in DC activation, we analyzed PKM2 expression in DCs, and we observed that PKM2 is increased at mRNA levels upon lipopolysaccharide (LPS)/IFNγ stimulation. Moreover, at mRNA level, TEPP-46 reduces the expression of pro-inflammatory markers upon LPS/IFNγ stimulation of DCs and inhibits the production of IL-12 and TNFα pro-inflammatory cytokines, as measured by ELISA. In addition, FACS analysis indicated that PKM2 activator seems to drive a reduced expression of the pro-inflammatory surface markers, CD40, CD80, CD86, and MHCII, by DCs upon LPS/IFNγ but did not promote the up-regulation of anti-inflammatory markers. Our preliminary data suggest that metabolic reprogramming of DCs through PKM2 tetramerization could reduce their pro-inflammatory activation.

## miR-142-3p Regulates TNF-Mediated Synaptopathy in Multiple Sclerosis

Silvia Caioli ^1,^*, Sara Balletta ^2^, Francesca De Vito ^1^, Alessandra Musella ^3^, Diego Fresegna ^4^, Valentina Vanni ^4^, Livia Guadalupi ^2^, Francesca Romana Rizzo ^2^, Krizia Sanna ^2^, Antonietta Gentile ^4^, Diego Centonze ^1^ and Georgia Mandolesi ^3^

^1^ Unit of Neurology, Istituto Neurologico Mediterraneo (INM) Neuromed-IRCCS, Pozzilli, Italy
^2^ Department Systems Medicine, University Tor Vergata, Rome, Italy
^3^ Department of Human Sciences and Quality of Life Promotion, University of Rome San Raffaele, Rome, Italy
^4^ Synaptic Immunopathology Lab, IRCCS San Raffaele Pisana, Rome, Italy
* Correspondence: silviacaioli@yahoo.it

Multiple sclerosis (MS) is a chronic inflammatory demyelinating disease of the central nervous system (CNS) triggered by an aberrant immune response against myelin. Recent preclinical and clinical studies have demonstrated that diffuse synaptic dysfunction and loss, known as excitotoxic synaptopathy, are a hallmark of MS pathophysiology. Proinflammatory cytokines, like TNF and IL-1β, contribute to the neuronal excitotoxic damage also by inducing synaptopathic small noncoding RNAs (miRs) in both MS and its mouse model, the experimental autoimmune encephalomyelitis (EAE). MiRs are new modulators of gene expression circulating in the cerebrospinal fluids (CSF), which have been recently proposed as diagnostic and prognostic biomarkers for MS. Specifically, we observed that miR-142-3p is increased in the CSF of MS patients as well as in cerebellum of EAE mice, where it causes synaptopathy-driven excitotoxic damage. Moreover, high miR-142-3p levels associate with a worse disease progression and therapeutical response. Coherently, miR-142 knock-out mice are totally resistant to EAE. In this research, we used transgenic heterozygous miR-142 mice as a good tool to investigate miR-142-3p role in EAE striatal synaptopathy since they show reduced miR expression in both the CNS and in the periphery compared to wild-type mice. By performing electrophysiological, immunohistochemical, and molecular experiments, we demonstrated that low miR-142-3p levels provide a full protection from TNF-driven synaptopathy in the presence of EAE striatal neuroinflammation and EAE symptoms. Furthermore, in a cohort of MS patients, we found a positive correlation between TNF levels in CSF and MS progression, as for miR-142-3p. Interestingly, we observed that the patients with high CSF levels of both TNF and miR-142-3p show the most severe disease progression index, suggesting that TNF needs high miR-142-3p levels to exert its worst detrimental effects. Further mechanistic studies are still ongoing.

## In-Vitro Exposure to Cladribine, a Targeted Lymphocyte-Reducing Drug for Multiple Sclerosis, Affects the Expression, Phosphorylation Status, and Activity of Deoxycytidine Kinase in Activated T Cells

Federico Carlini ^1,^*, Paola Barboro ^1^, Camillo Rosano ^1^, Aldo Profumo ^1^, Nicole Kerlero de Rosbo ^2^ and Antonio Uccelli ^1^

^1^ Ospedale Policlinico San Martino, Sistema Sanitario Regione Liguria-IRCCS, Genova, Italy
^2^ DINOGMI, Università degli Studi di Genova, Genova, Italy
* Correspondence: carlinifederico3@gmail.com

Activation of cladribine (2CdA), a drug approved for multiple sclerosis, is driven by a high ratio of deoxycytidine kinase (dCK)/5′nucleotidase. In view of their high dCK content, lymphocytes are preferential target for 2CdA. We demonstrated that the 2CdA-induced apoptosis in stimulated T cells is correlated with enhanced dCK expression and activity. Up to 16 dCK phosphorylation sites have been described to date, but little is known about how they affect dCK activity. Our objective was to assess the differential composition of post-translational dCK isoforms in healthy donor T cells activated or not with anti-CD3/CD28 antibodies in presence/or absence of 2CdA. We used Phos-tag™ electrophoresis, which traps phosphorylated proteins, thereby reducing their migration according to their phosphorylation status. Cell lysates treated with alkaline phosphatase were used to define the control band corresponding to de-phosphorylated dCK, and this latter was much reduced in unstimulated cells. Lysates from activated T cells showed five separate areas of phosphorylated dCK isoforms. Areas were fewer in lysates from activated T cells exposed to 2CdA, with a profile that appeared specific to the treatment. As areas 4 and 5 were consistently observed in all samples tested and could be reliably measured, we focused our analysis on these two areas. Our data suggest that exposure to 2CdA results in a shifted composition of phosphorylated dCK isoforms, which might be related to the activity of the enzyme and thereby influence the susceptibility of activated T cells to the drug. Further analysis of dCK phosphorylation status and activity in lymphocytes from 2CdA-treated multiple sclerosis patients will help understand the impact of 2CdA on pathological immune responses related to central nervous system autoimmunity.

## In-Vitro Validation of miR-23a-3p and miR-181a-5p Targeting *SNAP-25*

Simone Agostini ^1,^*, Elisabetta Bolognesi ^1^, Roberta Mancuso ^1^, Franca Rosa Guerini ^1^ and Mario Clerici ^2^

^1^ Laboratorio di Medicina Molecolare e Biotecnologie, IRCCS Fondazione Don Gnocchi, Milano, Italy
^2^ Dipartimento di Fisiopatologia e dei Trapianti, Università degli Studi di Milano, Milano, Italy
* Correspondence: sagostini@dongnocchi.it

SNAP-25 protein is a key component of the SNARE complex, involved in synaptic vesicles fusion with plasma membranes and neurotransmitter release, fundamental for the neural plasticity. Our recent paper showed that the concentration of three specific miRNAs, namely miR-27b-3p, miR-181a-5p, and miR-23a-3p, are associated with a specific *SNAP-25* polymorphism (rs363050). Target prediction in-silico analysis showed that all the three miRNAs target *SNAP-25*, but the binding between these miRNAs and the 3′UTR region of *SNAP-25* mRNA was never demonstrated. For this reason, here we verified in vitro whether these three miRNAs are able to bind and to modulate the expression of *SNAP-25*. Co-transfection of Vero cell line with the miRNAs mimic or inhibitor and luciferase reporter plasmid containing SNAP-25 3′UTR showed that miR-181a-5p (*p* ≤ 0.01) and miR-23a-3p (*p* < 0.05) but not miR-27b-3p can modulate the luciferase signal, confirming the interaction of these two miRNAs with *SNAP-25* 3′UTR region. Next, human oligodendroglial cell line (MO3.13) was transfected with miR-181a-5p and miR-23a-3p, confirming that the two miRNAs are able to regulate the *SNAP-25* gene and protein expression. Interestingly, the two miRNAs modulate *SNAP-25* in an opposite way, as miR-181a-5p significantly increased (*p* < 0.0005), whereas miR-23a-3p decreased (*p* < 0.0005) its expression. In conclusion, these results verify for the first time that miR-181a-5p and miR-23a-3p can modulate the *SNAP-25* expression; considering the important role of SNAP-25 on synaptic function and plasticity and that its deregulation has been associated with different diseases (i.e., autism, psychiatric disorders, dementia, and sarcopenia), these data highlight the importance of studying these miRNAs as potential biomarkers or therapeutic targets.

## Generation of Human iPSC-Derived 3D Cortico-Motor Assembloids for Disease Modelling

Maria Cristina Benedetti ^1,^*, Alessandro Rosa ^1^, Valeria De Turris ^2^, Federico Salaris ^2^, Chiara D’Antoni ^2^ and Silvia Di Angelantonio ^2^

^1^ Dipartimento Scienze Biochimiche, Università La Sapienza, Roma, Italy
^2^ Istituto Italiano di Tecnologia, CLNS@ Sapienza, Roma, Italy
* Correspondence: mariacristina.benedetti@uniroma1.it

Movement is controlled by a wide network of nerve cells that involves all levels of the nervous system, from the cortex to the spinal cord. The complexity of this system makes it challenging to identify the etiopathology of diseases affecting the cortico-spinal motor pathways due also to the lack of appropriate human models. The use of human induced pluripotent stem cells (iPSCs) has made it possible to study single components controlling movement, alone or in combination. Furthermore, the development of 3D organoids has allowed to generate more complex and physiological tissue models. Recently, the generation of cortico-motor assembloids has shown that cortical neural projection could control muscle contraction via activation of motor neurons. The aim of this project was to reproduce this complex network by fusing human cortical organoids (hCOs) with neuromuscular organoids (NMOs). NMOs self-organize to reproduce neuromuscular junction, formed by both spinal cord and musculoskeletal cells. Using human iPSCs, we generated and characterized hCOs and NMOs by morphological and molecular analysis, showing their ability to recapitulate the complexity of cortical and NMJ system over time. Their combination will provide deeper insight into the descending pathways that generate movement in health and disease to better understand the contribution of each cell types to the altered phenotype. Thus, 3D assembloids can be used as a platform for disease modelling and drug screening in order to develop new therapeutic approaches. In particular, this system will be used for modelling GNAO1 disorder, a rare genetic disease affecting psychomotor development with high clinical heterogeneity. We are generating four iPSC lines individually carrying different mutations in the GNAO1 gene by CRISPR/Cas9 system. These lines will be used to generate assembloids in order to dissect molecular mechanisms underlying the disease heterogeneity.

## Biallelic Variants in LIG3 Cause a Novel Mitochondrial Neurogastro-Intestinal Encephalomyopathy

Francesca Bianco ^1,^*, Christian Bergamini ^2^, Chiara Diquigiovanni ^3^, Isabella Ceccherini ^4^, Valerio Carelli ^5^, Marco Seri ^3^, Nicholas Katsanis ^6^, Floor A. M. Duijkers ^7^, Mariko Taniguchi-Ikeda ^8^, Roberto De Giorgio ^9^ and Elena Bonora ^3^

^1^ DIMEVET, University of Bologna, Bologna, Italy
^2^ FABIT, University of Bologna, Bologna, Italy
^3^ DIMEC, University of Bologna, Bologna, Italy
^4^ IRCCS Istituto Giannina Gaslini, Genova, Italy
^5^ Programma di Neurogenetica, IRCCS Istituto delle Scienze Neurologiche di Bologna, Bologna, Italy
^6^ Center for Human Disease Modeling, Duke University, Durham, NC 27710, USA
^7^ Department of Clinical Genetics, University of Amsterdam, Amsterdam, The Netherlands
^8^ Institute for Comprehensive Medical Science, Fujita Health University, Aichi, Japan
^9^ Department of Morphology, Surgeryand Experimental Medicine, St. Anna Hospital, Ferrara, Italy
* Correspondence: francesca.bianco5@unibo.it

Mitochondrial encephalomyopathies can be characterized by leukoencephalopathy due to mitochondrial dysfunction and severe abnormality of gut motility, such as chronic intestinal pseudo-obstruction, an impairment of gut propulsion. Mitochondrial neurogastrointestinal encephalopathy (MNGIE) is caused by mutations in *TYMP* or *POLG* or mitochondrial DNA (mtDNA) itself, but a number of patients are still unresolved. We aimed to identify the genetic defects in seven patients from three independent families showing severe gut dysmotility and neurological abnormalities, including leukoencephalopathy, epilepsy, migraine, stroke-like episodes, and neurogenic bladder. None of the patients carried mutations in *TYMP*, *POLG,* or mtDNA. Whole exome sequencing was performed on the DNA extracted from peripheral blood. Dermal fibroblasts were obtained from patients’ and controls’ skin biopsies and grown in standard culture media. Functional *lig3* ablation in zebrafish was performed via morpholino analysis and/or CRISPR/Cas9 gene editing. We identified heterozygous variants in a new disease gene named *lig3*. The *LIG3* gene encodes the only mtDNA ligase and plays a pivotal role in mtDNA repair and replication. In-vitro assays in patient-derived cells showed a decrease in *lig3* protein levels and ligase activity. We demonstrated that the *lig3* gene defects affect mtDNA maintenance, leading to mtDNA depletion. A decrease in the number of myenteric neurons and increased fibrosis and elastin levels were the most prominent changes in the gut. Muscle pathology of decreased cytochrome c-oxidase (COX) staining was also observed. Disruption of *lig3* in zebrafish reproduced the brain alterations and impaired gut transit in vivo and was rescued by the wild-type human *lig3* isoform but not the mutant one. We identified biallelic variants in the *lig3* gene that result in a novel mitochondrial phenotype characterized by predominant gut dysmotility, leukoencephalopathy, and neuromuscular abnormalities.

## Nothobranchius Furzeri Organotypic Cultures: Towards a Model of Ex-Vivo Brain Aging

Letizia Brogi ^1,^*, Sara Bagnoli ^1^, Eva Terzibasi Tozzini ^2^ and Alessandro Cellerino ^1^

^1^ Scuola Normale Superiore, BIO@SNS, Pisa, Italy
^2^ Stazione Zoologica Anton Dohrn, SZN, Napoli, Italy
* Correspondence: letizia.brogi@sns.it

Organotypic culture of brain slices is an ex-vivo technique used to investigate long-term neuronal survival. Organotypic cultures maintain a three-dimensional organization and mimic the in-vivo development of cells and synapses. The absence of the blood-brain barrier allows direct access of small molecules to the culture. In addition, organotypic cultures allow to study the effects of age on brain in isolation without the influence of the systemic milieu. The ex-vivo model has been widely used in rodents for conducting molecular, pharmacological, and physiological studies. To our knowledge, no long-term culture system for fish brains is established. The short-lived annual fish *Nothobranchiusfurzeri* shows extremely short life span and accelerated expression of age markers, and a long-term culture system would enable the study of brain aging ex-vivo. We thus established organotypic cultures from brain slices of *N. furzeri*. The brains were extracted from MZCS-222 fish 5, 12, and 30 weeks after hatching, from which we cut 500-μm slices of various brain regions. The brain slices were incubated on porous membranes in an ad-hoc medium for at least five weeks. Slices were incubated with EdU for the first three days to label new-born cells. One week after EdU treatment, we observed neurogenesis in all slices, indicating that adult neurogenesis is retained ex-vivo even in slices from old fish as well as in vivo. In addition, we specifically tested the viability of noradrenergic neurons labelled with TH, and we observed that these neurons persist for at least five weeks in vitro. Our future aims are to prolong the culture period to test whether brain aging markers become expressed in vitro and finally test drugs and nutraceutical compound.

## Unravelling Combined RNA Interference and Gene Therapy in In-Vitro and In-Vivo Disease Models as a Potential Therapeutic Strategy for CMT2A

Roberta De Gioia ^1,^*, Alessia Anastasia ^1^, Monica Nizzardo ^2^, Linda Ottoboni ^1^, Matilde Contardo ^3^, Silvia Bono ^1^, Sabrina Salani ^1^, Valentina Melzi ^1^, Serena Pagliarani ^1^, Elena Abati ^3^, Nereo Bresolin ^2^, Giacomo Comi ^2^, Stefania Corti ^2^ and Federica Rizzo ^2^

^1^ IRCCS Fondazione Ca Granda, Ospedale Maggiore Policlinico, Milano, Italy
^2^ IRCCS Fondazione Ca Granda Ospedale Maggiore Policlinico, Università di Milano, Milano, Italy
^3^ Università di Milano, Milano, Italy
* Correspondence: robertadegioia1989@gmail.com

Charcot-Marie-Tooth type 2A (CMT2A) is an inherited sensory-motor neuropathy caused by missense mutations in the *MFN2* (Mitofusin2) gene. MFN2 mutations appear to induce the disease with a dominant-negative mechanism, where the wild-type MFN2 allele expression is negatively regulated by the mutant protein. Gene therapy for dominant inherited diseases uses RNA interference (RNAi) to selectively inhibit expression of the mutant allele, which results in a toxic protein. Since this approach can also reduce the expression of the wild-type functional allele, wild-type allele restoration in combination with mutant allele silencing could improve the therapeutic effects. Here, we propose this double strategy as a possible CMT2A new therapeutic approach. Indeed, we tested the effective silence of the endogenous MFN2 (both mutant and wild-type MFN2 alleles), and its replacement with an exogenous copy of the wild-type MFN2 gene in CMT2A human induced pluripotent stem cells (iPSCs)-differentiated motor neurons and in Mitocharc1, a mouse model of CMT2A. To evaluate the amelioration of the disease phenotype after this strategy, we analysed key motoneuronal features relevant to CMT2A, observing an enhancement in mitochondrial distribution and function and beyond in apoptotic and autophagic parameters. Our data confirm the feasibility of combined RNAi and gene therapy approach as potential therapeutic strategy for treating CMT2A and other similar genetic neurological disorders.

## The Role of LRRK2 G2019S on Synaptic Neurotransmission in Parkinson’s Disease

Angela Di Iacovo ^1,^*, Ludovica Iovino ^2^, Tiziana Romanazzi ^1^, Manan Bhatt ^1^, Raffaella Cinquetti ^1^, Laura Civiero ^2^, Elena Bossi ^1^ and Cristina Roseti ^1^

^1^ Departmentof Biotechnology and Life Sciences, University of Insubria, Varese, Italy
^2^ Department of Biology, University of Padua, Padova, Italy
* Correspondence: adiiacovo@studenti.uninsubria.it

Parkinson’s disease (PD) is a neurodegenerative syndrome characterized by the loss of dopaminergic neurons in the substantia nigra pars compacta, with consequent reduction of striatum projections. Recently, Leucine-rich repeat kinase 2 (LRRK2) has been discovered to play a role in both monogenic and sporadic forms of PD. Several LRRK2 mutations are observed in PD patients, and among them, the substitution Gly2019Ser is the most common. It has been reported that a gain of function of the mutated kinase activity affects synaptic transmission; in particular, it is known as an influence on the glutamatergic pathway. Conversely, the role of LRRK2 on GAB-Aergic transmission is poorly understood. In order to gain insights on the relation among the G2019S LRRK2 mutation and GABAA receptors functionality, we assessed electrophysiological experiments using microtransplantation technique. Membranes from mouse striatum tissues of LRRK2-associated PD model were injected into *Xenopus laevis* oocytes, and excitatory and inhibitory currents were characterized using two-electrode voltage clamp. In G2019S striatum tissues, we found that enhanced glutamate evoked currents, according to the hypothesis of altered glutamatergic transmission in PD disease. Interestingly, the data show a significant reduction of GABA evoked currents amplitude in the LRRK2 G2019S condition. The ratio of glutamatergic and GABAergic currents confirms the impact of mutated LRRK2 on excitatory/inhibitory imbalance in the pathological tissue. To investigate the cause of GABA current reduction, we tested whether chloride homeostasis was altered in oocytes injected with LRRK2 G2019S membranes. The results demonstrate that the reduction of GABA current amplitude was not associated with a change in GABA reversal potential (EGABA). In conclusion, our preliminary data show a reduced GABAergic transmission in LRRK2 G2019S mouse tissues, raising fundamental issues on the role of LRRK2 in the modulation of neurotransmission.

## MTCH2 Functionally Co-Operates with BID in Promoting Ca^2+^-Induced Neuronal Injury

Beatrice D’Orsi ^1,^*, Natalia Niewidok ^2^, Heiko Düssmann ^2^ and Jochen Prehn ^2^

^1^ CNR, Institute of Neuroscience, Pisa, Italy
^2^ Department of Physiology and Medical Physics, Royal College of Surgeons in Ireland, Dublin, Ireland
* Correspondence: beatrice.dorsi@in.cnr.it

The BH3 interacting-domain death agonist (BID) is a pro-apoptotic member of the Bcl-2 protein family. While proteolytic processing of BID links death receptor-induced apoptosis to the mitochondrial apoptosis pathway, we previously showed that full-length BID also translocates to mitochondria during Ca^2+^-induced neuronal cell death. Moreover, mitochondrial carrier homolog 2 (MTCH2) was identified as a mitochondrial protein that interacts with BID during cell death. We started our studies by investigating the effect of *Mtch2* silencing in a well-established model of Ca^2+^-induced mitochondrial permeability transition pore opening in non-neuronal HCT116 cells. We found that silencing of *Mtch2* inhibited mitochondrial swelling and the associated decrease in mitochondrial energetics, suggesting a pro-death function for MTCH2 during Ca^2+^-induced injury. Next, we explored the role of BID and MTCH2 in mediating Ca^2+^-induced injury in primary cortical neurons triggered by prolonged activation of NMDA glutamate receptors. Analysis of intracellular Ca^2+^ transients, using time-lapse confocal microscopy, revealed that neurons lacking *Bid* showed markedly reduced Ca^2+^ levels during the NMDA excitation period. These Ca^2+^ transients were further decreased when *Mtch2* was also silenced. Collectively, our data suggest that BID and MTCH2 functionally interact to promote Ca^2+^-induced neuronal injury.

## Transcriptome Analysis of miRNAs and Their Interactors in FTD Patients’ Small Extracellular Vesicles

Maria Garofalo ^1^, Francesca Dragoni ^1,^*, Daisy Sproviero ^2^, Eleonora Corridori ^3^, Alfredo Costa ^4^, Giulia Perini ^4^, Matteo Cotta Ramusino ^4^, Orietta Pansarasa ^2^ and Stella Gagliardi ^2^

^1^ Department of Biology and Biotechnology “L. Spallanzani,” University of Pavia, Pavia, Italy
^2^ Genomic and Post-Genomic Unit, IRCCS Mondino Foundation, Pavia, Italy
^3^ Centro di Eccellenza sulle Malattie Neurodegenerative, Dipartimento di Scienze Farmacologiche e Biomolecolari, University of Milan, Milano, Italy
^4^ Unit of Behavioral Neurology, IRCCS Mondino Foundation, Pavia, Italy
* Correspondence: francesca.dragoni@mondino.it

Extracellular vesicles (EVs) cargo has been evaluated in neurodegenerative disorders, especially concerning their microRNAs content. Fronto-temporal dementia (FTD) is characterized by aggregation of proteins (TDP-43 and Tau) in the frontal and temporal lobes with microvacuolation and relevant deregulation of RNA-binding proteins (RBPs). We investigated miRNA cargo of small EVs (SEVs) derived from plasma of FTD patients and healthy controls for evaluating deregulated miRNAs in patients to highlight new peripheral biomarkers. Moreover, we aimed to identify mRNA targets involved in FTD pathogenesis. SEVs were isolated from plasma of nine FTD patients and nine healthy volunteers by differential centrifugation and characterized by Nanosight. MicroRNA libraries were generated using Small RNA-Seq Library Prep Kit (Lexogen) and sequenced on a NextSeq 500/550 (Illumina). Interaction prediction was carried out on TarBase v.8 database. We found a total of 197 differentially expressed (log-fold change (FC) >1 and <−1) microRNAs, 99 up-regulated and 98 down-regulated. Then, we looked for directly validated mRNA targets of the most deregulate microRNAs (5 up and 5 down-regulated) in our analysis. Interestingly, hsa-miR-522-5p, down-regulated in our profiling, targets RTN3, which in turn interacts with and modulates BACE1, and the up-regulation of hsa-miR-203a-3p may have an impact on TNF and IL-12 levels, which were reduced in CSF of FTD patients. Moreover, hsa-miR-181c-5p was up-regulated, and its role was already linked to a negative feedback network of TDP43. We also evaluated the deregulation of microRNA already associated to other dementia types, and we found a down-regulated microRNA in common with Alzheimer’s disease, namely hsa-miR-1260b, involved in Wnt pathway. In conclusion, our data highlight the importance of microRNAs cargo examination in EVs of FTD patients. In fact, their potential is exploitable both for biomarkers discovery and for study of gene expression alteration in FTD pathogenesis.

## Exploiting Human Genetics of Multiple Sclerosis for Drug Repositioning as Antioxidant Redox Modifiers

Alessia Formato ^1,^*, Silvia Corbisiero ^1^, Maristella Steri ^2^, Stefania Olla ^2^ and Cristina Agresti ^1^

^1^ Istituto Superiore di Sanità, Neuroscience, Rome, Italy
^2^ National Research Council, Istituto di Ricerca Genetica e Biomedica, Cagliari, Italy
* Correspondence: aformato92@gmail.com

Multiple sclerosis (MS) is the most common chronic inflammatory and progressively disabling disease of the central nervous system in young adults, characterized by demyelination, oligodendrocyte loss, and neuroaxonal degeneration in the white and gray matter of the brain and spinal cord. There is significant evidence that the sustained inflammatory phase of MS creates an imbalance between Reactive Oxygen and Nitrogen Species generation and the antioxidant defense systems causing oxidative/nitrosative stress (OS/NS), which has a potential role in MS-specific damage. Thus, the development of drugs able to effectively support the maintenance of redox homeostasis represents a rational approach to treat this disease. Our idea is to identify genes and/or gene products involved in MS that are linked to antioxidant pathways and repurposable drugs acting as modulators of these targets. To this aim, first we identified 698 different MS genetic variants from the Genome-Wide Association Studies (GWAS) Catalog. To assign the most reliable gene target to each associated variant, molecular Quantitative Trait Loci (QTLs) were searched for each hit in public databases. In parallel, we selected 22 OS-related pathways by the Reactome database and extracted all possible proteins that have been successively overlapped with MS genetic results. Among the 91 common targets identified, we selected gene targets that are known to be modulated by at least one approved or in clinical trial drug by means of four different drug databases. With the purpose of selecting the best drugs, we performed in-silico ADME-Tox (Absorption Distribution, Metabolism, Excretions, and Toxicology) studies, assigning a higher priority to orally administrable compounds expected to cross the blood-brain barrier. This strategy could promote the development of successful regenerative therapies for MS using existing drugs capable of modulating biomarkers of OS.

## miR-29a Is Modulated by One-Carbon Metabolism and Involved in Neurodegeneration

Tiziana Raia *, Daniele Antinori, Mariano Bizzarri, Marco Lucarelli and Andrea Fuso

Deptartment of Experimental Medicine, Sapienza University, Rome, Italy
* Correspondence: tiziana.raia@uniroma1.it

Alzheimer’s disease (AD) is a neurodegenerative disease and the most common cause of dementia in the elder population. It is characterized by the loss of neurons involved in cognitive functions due, among other factors, to the accumulation of beta-amyloid (Aβ) which, in turn, could be due to the loss of epigenetic control in the expression of genes involved in AβPP (amyloid-β protein precursor) processing. Some of these genes are controlled by their promoters methylation, a process related to “one carbon metabolism,” leading to the production of S-adenosyl-methionine (SAM), the main endogenous methyls donor. microRNAs (miRNAs) are associated to several diseases, including AD. To investigate if the modulation of the methylation pathway induced by B-vitamin deficiency and SAM-supplementation could change their expression, miRNAs were assessed by total RNA extraction, specific retrotranscription, and Real-time PCR. miR-29a was selected for its involvement in methylation processes and AD after a screening in human SK-N-BE neuroblastoma cells, cultured in control and B-deficient medium with or without SAM-supplementation. miR-29a was also analyzed in mice (under the described B-deficient and SAM-supplemented conditions) and in brain samples from healthy subjects and patients. Then we studied in vitro the effects of miR-29a silencing/over-expression by assessing its specific targets. miR-29a was repressed in B-deficiency (hypomethylation), over-expressed with SAM (hypermethylation) both in cells and in mice brain and down-regulated in post-mortem AD brains. This demonstrates that miRNAs expression is associated to DNA-methylation both directly and indirectly, suggesting that one carbon metabolism can interfere with the AD pathogenesis not just through gene-specific methylation. miR-29a silencing/over-expression experiments have the purpose of evaluating its role in AD models and use as an epigenetic biomarker and a new treatment approach to the disease.

## Patient-Derived 3D Glioblastoma-Culture Models: Characterization and Potential Applications in Drug Screening

Alessia G. Bosio *, Federica Barbieri, Adriana Bajetto, Alessandra Pattarozzi and Tullio Florio

Sezione di Farmacologia-Dipartimento di Medicina Interna, Università di Genova, Genova, Italy
* Correspondence: alessiagraziana.bosio@edu.unige.it

In the last decades, glioblastoma (GBM) therapy, despite remarkable research efforts, has not achieved significant improvement in patient survival, also due to the lack of appropriate models to study the role of cellular heterogeneity and microenvironment in its growth and invasiveness. 2D cultures of patient-derived cancer stem cells (CSCs), one of the key players in GBM relapse, have been fundamental to deeply study GBM development and drug resistance. However, this model lacks mimicking cell-cell and cell-microenvironment interactions. Recently, several 3D culture models have been developed to overcome the weaknesses of monolayer cultures. Here, we present a 3D culture model obtained from patient-derived CSCs embedded in Matrigel, a mouse sarcoma-derived extracellular matrix. In these conditions, GBM CSCs organized themselves in a tissue-like structure and continued to grow for more than 30 days. We identified a spatial localization of proliferating and Sox2+ and Olig2+ cells (likely CSCs) in the external layer, while non-proliferating and GFAP+ cells (differentiated GBM cells) were in the inner region of spheroids. By qRT-PCR, we showed that 3D growth induced CD44 expression, which was low in monolayer cultures. Metformin and novel biguanide analogues reduced proliferation rate in 3D as well as 2D cultures. Lastly, we developed a new 3D model in which minced GBM specimens are embedded in Matrigel (tumoroids). In these conditions tumor fragments are able to grow for several months and invade the surrounding matrix. By immunofluorescence, we identified βIII-tubulin, Sox2, GFAP, Olig2, CD31, and IBA1 positive cells within tumoroids, indicating that these culture conditions maintain the viability of different cell subpopulations composing GBM. In conclusion, 3D culture models, both from CSCs or minced GBM specimens, recapitulate the in-vivo cell-cell interaction and tumour cell hierarchy and could represent a model for drug screening with high translational validity.

## Early Evidence of Reduced Myelination in the Cortex of a Mouse Model of CDKL5 Deficiency Disorder

Sunaina Devi ^1,^*, Antonia Gurgone ^1^, Debora Comai ^1^, Riccardo Pizzo ^1^, Chiara Salio ^2^, Martina Lorenzati ^3^, Annalisa Buffo ^3^ and Maurizio Giustetto ^1^

^1^ Departmentof Neuroscience, University of Turin, Torino, Italy
^2^ Departmentof Veterinary Sciences, University of Turin, Torino, Italy
^3^ NICO-Department of Neuroscience, University of Turin, Torino, Italy
* Correspondence: sunaina.devi@unito.it

CDKL5 deficiency disorder (CDD) is a rare neurodevelopmental disease without a cure that is caused by mutations in the gene cyclin-dependent kinase-like 5 (*CDKL5*), and it is characterised by early-onset epilepsy as well as severe cognitive, sensorimotor, and intellectual disabilities. CDKL5 is a serine/threonine kinase that is expressed early during postnatal development in neurons, where it phosphorylates epigenetic factors (MeCP2, DNMT1), elements of both axonal and dendritic compartment (Shootin1, NGL1), and microtubule-associated proteins (MAP1S, EB2), which are crucial in nucleation and assembly of microtubules (MT). Along with neurons, CDKL5 is also expressed in glial cells, including oligodendrocytes (OL) and OL precursor cells (OPCs), underlying the myelination process. Although growing evidence indicate that the organization of myelin sheath can be severely compromised in the autism spectrum and in other neurodevelopmental disorders, whether myelin is affected by *CDKL5* mutation is still unknown. To start addressing this issue, we evaluated the extent of myelination, its developmental trajectory, and the expression of molecules modified by myelin deposition or axonal injury, i.e., MBP (myelin basic protein) and NF (neurofilaments), in *Cdkl5* KO mice, an established model of CDD. By using both immunofluorescence and western blot analysis of young (P15) and adult mice (P56), we found that mutant mice show a reduction of both MBP and phospho-NFs expression in primary somatosensory and visual cortices compared to WT animals, whereas no changes were detected in total NFs expression. The analysis of myelinated axons using transmission electron microscopy showed that the g-ratio was increased in mutants indicating that myelin sheath in CDKL5 KO mice is reduced compared to controls. In conclusion, our data indicate that cortical areas in CDD animals exhibit a global reduction/distortion of myelination, thus disclosing a novel role of *Cdkl5* in the CNS.

## The Neurobehavioral Protective Effect of Beta-D-Glucan Dietary Supplementation in a Murine Model of Obesity and Psychosocial Stress

Carlotta Baroni ^1,^*, Cristina Spalletti ^2^, Jacopo Agrimi ^1^, Matteo Caleo ^2^ and Vincenzo Lionetti ^1^

^1^ Istituto di Scienze della Vita, Scuola Superiore Sant’Anna, Pisa, Italy
^2^ Consiglio Nazionale delle Ricerche, Istituto di Neuroscienze, Pisa, Italy
* Correspondence: c.baroni4394@gmail.com

Lifestyle-related risk factors, mainly obesity and work-related stress, hugely affect brain health, contributing to the onset of serious diseases, such as stroke, dementia, and mood and anxiety disorders. The in-depth study of the mechanisms that trigger these pathogenetic processes, aimed to identify prevention and treatment strategies, requires a translational animal model capable of mimicking dysfunctions and helping validate neuroprotective non-invasive interventions, such as nutraceuticals compounds. To fill this gap, a murine neurobehavioral dysfunction model (NDM) was developed by subjecting 10-week-old C57BL/6J mice to high-fat diet (HFD) for 18 weeks and psychosocial stress (PS) via Resident Intruder Paradigm during the last two weeks of diet. Behavioral assessment was carried out through Y-maze and Elevated Plus Maze, while morpho-functional analyses were performed on perfused hippocampal slices. The NDM characterization highlighted hippocampal remodeling and Brain-Derived Neurotrophic Factor depletion. HFD+PS animals also exhibited anxiety-related traits, depressive-like behaviour, and spatial memory decay. The effect of a nutraceutical treatment was evaluated by adding 3% of barley Beta-D-Glucan (βGlucan) from the eighth week of HFD. The βGlucan-treated mice (HFDβ+PS) were compared with the NDM (HFD+PS) and with the control group, fed with standard diet and not stressed (SD). βGlucan determined spatial memory recovery and normalization of anxiety-related traits in the HFDβ+PS group. Of note, hippocampal immunohistochemistry found levels of synaptic plasticity (PV+interneurons), neurogenesis (BrdU+cells), and astrogliosis (GFAP) comparable to the SD group and statistically different from HFD+PS mice, while dentate gyrus volume (Hoechst) was not restored in the treated animals. Overall, our data reveal the neuroprotective activity exerted by βGlucan in subjects at high cerebrovascular risk and that the NDM has a high valuable potential to be employed for future investigations.

## Neurophysiological Investigation of Numerosity Adaptation

Irene Petrizzo *, Paolo Antonino Grasso, Giovanni Anobile and Roberto Arrighi

Dipartimento di Neuroscienze, Psicologia, Università degli Studi di Firenze, Area del Farmaco e Salute del Bambino, Firenze, Italy
* Correspondence: irene.petrizzo1@gmail.com

Visual adaptation is a phenomenon that occurs whenever the prolonged presentation of a stimulus biases the perception of the subsequent ones. This property of the visual system has been known for a long time (since the report of the waterfall illusion), and it is often exploited in psychophysical research to investigate brain perceptual mechanisms. One of the latest visual features that has been reported to be susceptible to adaptation is stimulus numerosity. As a consequence of a sustained exposure to a highly numerous array of dots, a subsequent array is grossly underestimated (and vice versa). Despite the robustness of this perceptual illusion (changes in perceived numerosity up to 50%), the brain mechanisms responsible for numerosity adaptation are still far from being fully understood. In the present study, we attempted to fill in this gap by leveraging on an electrophysiological approach. Previous evidence has found that non-symbolic numerosity perception modulates both early (N1) and late (P2p) components of the EEG signal. Here, we applied EEG to a well-established numerosity adaption paradigm to investigate whether short-term numerosity plasticity relies on the early or late component of numerical processing. Subjects were required to perform an active numerosity estimation task both in neutral and high-numerosity adapting conditions. To prevent any possible effect induced by other factors, non-numerical characteristics of the stimuli were carefully controlled for. Our preliminary results revealed a modulation of P2p induced by highnumerosity adaptation, with this effect being more prominent for stimuli presented in the left hemifield. Overall, these results suggest that numerosity adaptation is a phenomenon occurring at the later processing stages of the visual hierarchy, likely involving visual areas beyond the primary visual cortex.

## A Whole-Brain Approach to Map the Individual Impact of Gliomas on Brain Function

Erica Silvestri ^1,^*, Manuela Moretto ^2^, Silvia Facchini ^3^, Diego Cecchin ^4^, Maurizio Corbetta ^3^ and Alessandra Bertoldo ^1^

^1^ Department of Information Engineering, University of Padova, Padova, Italy
^2^ Padova Neuroscience Center, University of Padova, Padova, Italy
^3^ Department of Neuroscience, University of Padova, Padova, Italy
^4^ Unit of Nuclear Medicine, Department of Medicine, University of Padova, Padova, Italy
* Correspondence: erica.silvestri@unipd.it

Neurosurgical resection is the first-line therapeutic approach to the treatment of brain tumors, and a gross total resection is associated with a prolonged survival. Nevertheless, the benefits of a larger resection must be balanced against the risks of significant decrements of the quality of life. Current strategies employ intra-operative stimulation or specific task/rest state functional mapping to delineate areas to be preserved. One main limitation of these approaches is that they overlook distal regions or networks that could be functionally impaired by the tumor. Here, we developed a novel method to detect whole-brain resting-state networks (RSNs) alterations at the individual level for a safer surgery planning; then, we applied such method to investigate the impact of brain gliomas on RSNs. Functional and structural images of 28 patients with de-novo gliomas (13F/15M; age 59.6 ± 15.8 y) were acquired on a 3T Siemens Biograph mMR scanner at the University Hospital of Padova. From a publicly available dataset of controls (HCs), we derived a high-resolution RSNs template using independent component analysis (ICA) and extracted the same RSNs at the patient level by means of the group-information-guided ICA. Next, the alteration of single RSNs were detected computing the cosine similarity between the spatial map of each patient’s RSN as compared to a group of HCs. Moreover, we calculated the overlap between altered RSN maps and the tumour extent to investigate the spatial relationship between altered RSNs and tumour location. Tumours caused broad alterations of RSNs topography that occurred mainly in structurally normal regions outside of the tumor and edema region. On the contrary, cortical regions near the tumor often showed normal synchronization. Gliomas have a critical functional impact on remote regions and networks. A whole-brain functional mapping approach entirely performed at rest could provide helpful information for tumour surgery planning.

## Investigating the Impact of Different Free Surfer Approaches on Cortical Thickness Estimation

Giulia Debiasi *, Ilaria Mazzonetto and Alessandra Bertoldo

Department of Information Engineering, University of Padova, Padova, Italy
* Correspondence: giulia.debiasi.1@phd.unipd.it

Cortical thickness is one of the most important structural parameters. Among a variety of other anatomical features, it can be successfully used to study the relationship between cortical anatomy, disease, and cognition. One of the most common tools for the automatic estimation of the cortical thickness is FreeSurfer, now available in versions 6 (FS6) and 7 (FS7). This software requires as input a T1-weighted (T1w) image and, when available, a T2-weighted (T2w) image. To the best of our knowledge, no studies have investigated the impact of the different versions or the use of different inputs on cortical thickness estimation. In order to do so, T1w and T2w images from 129 minimally processed healthy subjects (68/61 M/F, from 36 to 86 years old) from the HCP-Aging project were submitted to the FreeSurfer main pipeline. Four different approaches were implemented: FS6 with T1w (FS6T1), FS6 with T1w and T2w (FS6T1T2), FS7 with T1w (FS7T1), and FS7 with T1w and T2w (FS7T1T2). Cortical thickness was extracted from 148 regions of interest according to the Destrieux atlas. Statistical differences between the approaches were evaluated by a two-way repeated measures ANOVA and post-hoc tests. Results show significant effects (*p* < 0.001) due to software version (FS6 vs. FS7, F1 = 127.1) and images used (T1w vs. T1wT2w, F1 = 779.4) in cortical thickness estimation. Post-hoc tests (*p* < 0.01 after correction for multiple comparisons) reveal that (i) adding the T2w image as input of the pipeline increases cortical thickness value and (ii) results obtained with FS6 are higher than FS7 ones. Our study proves that different approaches bring to different cortical thickness estimations. Therefore, when comparing values of cortical thickness from different studies, it is important to consider both the software version and the images used.

## A Connectomic Approach to Investigate Structural Brain Connectivity in Fabry Disease

Ilaria Gabusi ^1,^*, Matteo Battocchio ^1^, Sara Bosticardo ^1^, Arturo Brunetti ^2^, Sirio Cocozza ^2^, Alessandro Daducci ^1^, Antonio Pisani ^3^, Simona Schiavi ^4^ and Giuseppe Pontillo ^2^

^1^ Department of Computer Science, University of Verona, Verona, Italy
^2^ Department of Advanced Biomedical Sciences, University of Naples “Federico II,” Naples, Italy
^3^ Department of Public Health, University of Naples “Federico II,” Naples, Italy
^4^ Department of Neuroscience, Rehabilitation, Ophthalmology, Genetics, Maternal and Child Health (DINOGMI), University of Genoa, Genoa, Italy
* Correspondence: ilaria.gabusi@univr.it

Fabry disease (FD) is a rare and progressive systemic X-linked lysosomal storage disorder. With reference to central nervous system involvement, widespread microstructural alterations affecting the normal appearing white matter have been demonstrated. We analyzed brain structural connectivity of 46 FD patients (28F, 42.2 ± 13.2 y) and 49 healthy controls (HC, 21F, 42.3 ± 16.3 y). Diffusion-weighted magnetic resonance images were processed using probabilistic tractography and Convex Optimization Modeling for Microstructure Informed Tractography (COMMIT). To build quantitative connectomes, we employed a modified Automated Anatomical Labeling parcellation with 100 regions and weighted each connection by the total signal fraction associated to the corresponding bundle of streamlines. From each brain network, we extracted five global metrics: density (as an index of how much the network is connected), mean strength (showing how much these connections are strong), global efficiency (as a measure of the ability to exchange information), clustering coefficient (indicating how well a node is connected to its neighbors), and modularity (showing the degree of segregation). Between-group comparisons were performed by means of robust linear model, considering age and gender as confounding factors. We found statistically significant differences in both global efficiency (*p* = 0.01) and mean strength (*p* < 0.001) distributions. These results confirmed the mild but widespread microstructural damage occurring in FD, which reduces the effectiveness of fiber connections to efficiently exchange information. Moreover, since, for a subset of 11 subjects, neuropsychological tests were available, correlations between them and the adjusted global metrics were probed to evaluate the clinical impact of these structural changes. Significant correlation emerged between the mean strength and Rey Auditory Verbal Learning Test score (r = 0.721, *p* = 0.03), further highlighting the relevance of these findings.

## Mitochondrial Impairment in Lymphoblasts Derived from Aicardi-Goutières Syndrome’ Patients

Francesca Dragoni ^1,^*, Jessica Garau ^2^, Daisy Sproviero ^2^, Silvia De Siervi ^2^, Simona Orcesi ^3^, Orietta Pansarasa ^2^, Stella Gagliardi ^2^ and Cristina Cereda ^2^

^1^ Department of Biology and Biotechnology “L. Spallanzani,” University of Pavia, Pavia, Italy
^2^ Genomic and Post-Genomic Unit, IRCCS Mondino Foundation, Pavia, Italy
^3^ Child and Adolescent Neurology Unit, IRCCS Mondino Foundation, Pavia, Italy
* Correspondence: francesca.dragoni@mondino.it

Aicardi-Goutières Syndrome (AGS) is an uncommon childhood disease affecting the brain, immune system, and skin. Nine AGS gene mutations cause a buildup of endogenous nucleic acids (NAs) that the organism misidentifies as foreign NAs of viral origin, resulting in an aberrant Interferon-alpha (IFN-)-mediated immunological response. Mitochondrial abnormalities can result in the release of mtDNA and the production of IFN-, which can stimulate immunological pathways. The aim of this work was to examine mitochondria in AGS patient cells and determine if they play a role in the disease’s pathogenesis. The study employed lymphoblasts (LCLs) from *RNASEH2A* and *RNASEH2B* mutant AGS patients and one healthy control. The morphological alterations, ROS generation, and membrane potential changes of these organelles were studied by transmission electron microscopy and flow cytometry. The SeaHorse Analyzer was used to investigate the metabolic changes, while immunofluorescence was utilized to study mtDNA oxidation and the VDAC oligomerization. The release of mtDNA was investigated using qRT-PCR. Both mutant LCLs showed morphological and structural changes in mitochondria when compared to controls, while *RNASEH2A* LCLs showed a lack of physiologic membrane potential. ROS generation was enhanced in both mutant LCLs although it was considerably greater in *RNASEH2B* LCLs. According to these findings, *RNASEH2B* LCLs had a higher levels of 8-oxoG, a marker of mtDNA oxidation, and a stronger signal generated from VDAC protein oligomerization, indicating the development of a mitochondrial pore that might enable the release of mtDNA into the cytoplasm. In *RNASEH2B* LCLs, there was a significant increase in cytoplasmic mtDNA content. The damage to these structures in both mutant cell lines was also verified by metabolic changes. Our findings support the existence of mitochondrial abnormalities in LCLs from AGS patients, indicating that these organelles could be involved in the disease’s etiology.

## Multi-OMICs Approach to Elucidate the Role of Candidate Modifiers in C9orf72-ALS Patient-Derived Spinal Cord Organoids

Noemi Galli ^1,^*, Benedetta Frizzi ^2^, Irene Faravelli ^1^, Mafalda Rizzuti ^1^, Gianluca Costamagna ^3^, Fabio Biella ^3^, Manuela Garbellini ^3^, Giulia Garrone ^4^, Monica Nizzardo ^1^ and Stefania Corti ^1^

^1^ Centro Dino Ferrari, Unità di Neuroscienze, Dipartimento di Fisiopatologia Medicochirurgica e dei Trapianti (DEPT), Università degli Studi di Milano, Milano, Italia
^2^ Dipartimento di Fisiopatologia Medico-Chirurgica e dei Trapianti, Università Degli Studi di Milano, Milano, Italia
^3^ Ospedale Maggiore Policlinico di Milano, Fondazione IRCCS Ca’ Granda, Milano, Italia
^4^ UNITECH OMICS Platform, Università degli Studi di Milano, Milano, Italia
* Correspondence: gallinoemi.994@gmail.com

Amyotrophic lateral sclerosis (ALS) is a rare neuronal disorder involving motor system that is characterized by upper and lower motor neuron (MN) loss, resulting in progressive muscle paralysis that eventually costs the life of the patient. No therapy is able to effectively slow, halt, or reverse disease progression. Therefore, expanding our knowledge of ALS pathophysiology is imperative to develop novel treatment strategies. 3D models represent a challenging tool that can recapitulate the complexity of tissue framework, unlike limited 2D cultures. The primary goal of this proposal was to (1) characterize spinal cord organoids to maximize their reproducibility and reliability; (2) outline the ALS phenotype in this model with omic approaches; and (3) identify and validate candidate genes/pathways related to the pathophysiology of the disease.

On this basis, we generated spinal cord organoids, and we verified the presence of neural progenitors, post-mitotic neurons, MNs, and glia. Organoids were harvested, fixed, and cryosectioned at three timepoints (day 30, 45, and 80) and evaluated for their morphology and neurodevelopmental features by IHC and qPCR. Specifically, day-80 organoids expressed markers, such as MAP2, DCX, OLIG2, PAX6, SMI32, TUBB3, and GFAP. Terminally differentiated spinal cords underwent mass spectrometry to reveal proteins differentially expressed in ALS samples compared to controls. We reported around 250 significantly dysregulated proteins that we are currently validating with western blot and qPCR. Preliminary gene ontology analysis depicted alterations associated with cytoskeleton, energy metabolism, and astrocyte reactivity. Finally, organoids at day 80 were dissociated and cryopreserved to investigate possible aberrant transcriptomic profile related to ALS pathogenesis through single-cell RNA sequencing.

Overall, this project might allow the assessment of novel candidate genes linked with C9orf72-ALS pathogenesis and their potential as therapeutic targets.

## LRRK2-Related Parkinson’s Disease Mutation Impairs Glutamate Trans-Porter Trafficking

Ludovica Iovino ^1,^*, Veronica Giusti ^1^, Francesca Pischedda ^2^, Elena Giusto ^3^, Nicoletta Plotegher ^1^, Antonella Marte ^4^, Ilaria Battisti ^5^, Angela Di Iacovo ^6^, Algerta Marku ^7^, Giovanni Piccoli ^2^, Rina Bandopadhyay ^8^, Carla Perego ^7^, Tiziana Bonifacino ^9^, Giambattista Bonanno ^10^, Cristina Roseti ^6^, Elena Bossi ^6^, Giorgio Arrigoni ^5^, Luigi Bubacco ^1^, Elisa Greggio ^1^, Sabine Hilfiker ^11^ and Laura Civiero ^12^

^1^ Department of Biology, University of Padova, Padova, Italy
^2^ Department of Cellular, Computational and Integrative Biology—CIBIO, University of Trento, Trento, Italy
^3^ IRCCS, San Camillo Hospital, Venezia, Italy
^4^ Department of Experimental Medicine, University of Genova, Genova, Italy
^5^ Department of Biomedical Sciences, University of Padova, Padova, Italy
^6^ Center for Research in Neuroscience, Department of Biotechnology and Life Sciences, University of Insubria, Varese, Italy
^7^ Department of Pharmacological and Biomolecular Sciences, University of Milano, Milano, Italy
^8^ UCL Queen Square Institute of Neurology, Reta Lila Weston Institute of Neurological Studies, London, UK
^9^ Department of Pharmacy-DIFAR, University of Genova, Genova, Italy
^10^ Department of Pharmacy-DIFAR/Ospedale Policlinico San Martino, University of Genova/IRCCS, Genova, Italy
^11^ Department of Anesthesiology, Physiology, Pharmacology, and Neuroscience, Rutgers New Jersey Medical School, Newark, NJ, USA
^12^ Department of Biology/San Camillo Hospital, University of Padova/IRCCS, Paodva/Venezia, Italy
* Correspondence: ludovica.iovino@unipd.it

The Excitatory Amino Acid Transporter 2 (EAAT2) accounts for 80% of brain glutamate clearance and is mainly expressed in astrocytic perisynaptic processes. EAAT2 function is finely regulated by endocytic events, recycling to the plasma membrane, and degradation. Noteworthy, deficits in EAAT2 have been associated with neuronal excitotoxicity and neurodegeneration. In this study, we show that EAAT2 trafficking is impaired by the leucine-rich repeat kinase 2 (LRRK2) pathogenic variant G2019S, a common cause of late-onset familial Parkinson’s disease (PD). In LRRK2 G2019S human brains and experimental animal models, EAAT2 protein levels are significantly decreased, which is associated with elevated gliosis. The decreased expression of the transporter correlates with its reduced functionality in mouse LRRK2 G2019S purified astrocytic terminals and in *Xenopus laevis* oocytes expressing human LRRK2 G2019S. In Lrrk2 G2019S knockin mouse brain, the correct surface localization of the endogenous transporter is impaired, resulting in its interaction with a plethora of endo-vesicular proteins. Mechanistically, we report that pathogenic LRRK2 kinase activity delays the recycling of the transporter to the plasma membrane, causing its intracellular relocalization and degradation. Taken together, our results demonstrate that pathogenic LRRK2 interferes with the physiology of EAAT2, pointing to extracellular glutamate overload as a possible contributor to neurodegeneration in PD.

## miRNAs Shuttled by Exosomes Produced by Mesenchymal Stem Cells Ameliorate the Astrocyte Phenotype in Late Symptomatic SOD1G93A Mouse Astrocyte Primary Cultures

Mandeep Kumar ^1,^*, Francesca Provenzano ^1^, Debora Giunti ^2^, Marco Milanese ^1^, Benedetta Parodi ^2^, Nicole Kerlero Rosbo ^2^, Antonio Uccelli ^3^ and Giambattista Bonanno ^1^

^1^ Department of Pharmacologyand Toxicology, University of Genova, Genova, Italy
^2^ Department of Neurosciences, Ophthalmology, University of Genova, Genetics, Rehabilitation and Child Health, Genova, Italy
^3^ IRCCS, San Martino Polyclinic Hospital, Genova, Italy
* Correspondence: mandeep.pharm@gmail.com

Amyotrophic lateral sclerosis (ALS) is a fatal neurodegenerative disease characterized by muscle wasting, weakness, and spasticity due to progressive degeneration of cortical and spinal motor neurons. Both familiar and sporadic ALS have been described. Despite the notable growth of genetic studies, the genesis of most sporadic cases remains unknown. Currently, epigenetic research involving miRNA studies shows some promising aspects.

We previously reported that intravenous administration of mesenchymal stem cells (MSCs) in the SOD1G93A ALS mouse model significantly improved disease progression and modulated the astrocyte and microglia reactive phenotype. We proposed that MSC effects were paracrine, possibly involving exosome-mediated cell communication. Indeed, unpublished results substantiate the positive impact of MSC-derived exosomes on spinal cord primary astrocyte cell cultures prepared from late symptomatic 120-day-old SOD1G93A mice.

Here, we investigated the effects of nine miRNA, which were found up-regulated in IFNɣ-primed MSCs and shuttled by MSC-derived exosomes. For this purpose, we transfected SOD1G93A astrocytes with the single synthetic miRNAs and analysed their effect on the astrocyte phenotype. Seven out of nine miRNA mimics significantly decreased the overexpression of GFAP, IL1β, and TNFα detected by confocal microscopy in SOD1G93A astrocytes.

Four of these miRNAs (466q, 467f, 466m5p, and 466i3p) were over-expressed in MSCs and in exosomes. We selected in silico their relevant pathways (p38, TNFα, and NFKB) that have been validated by determining the miRNA effects on MAP3K8, MAPK-APK2, MAPK11, and TRAF6 by qPCR. 466q and 467f strongly reduced MAPK11 mRNA expression, thus inhibiting TNFα formation.

Our results suggest that the amelioration of the reactive phenotype of spinal cord SOD1G93A astrocytes, brought about by in-vivo MSC treatment, operates through exosome-shuttled specific miRNAs.

## Dissecting the Molecular Mechanism linking GCase Deficiency with the Onset of Neurodegeneration in Gaucher and Parkinson’s Diseases

Giulia Lunghi ^1,^*, Emma Veronica Carsana ^1^, Samarani Maura ^2^, Emanuele Frattini ^3^, Rosaria Bassi ^1^, Nicoletta Loberto ^1^, Alessio Di Fonzo ^3^ and Massimo Aureli ^1^

^1^ Department of Medical Biotechnology and Molecular Medicine, University of Milano, Segrate, Italy
^2^ Department of Cell Biology and Infection, Institute Pasteur, Paris, France
^3^ Movement Disorder Research Group, Fondazione IRCCS Cà Granda Ospedale Maggiore Policlinico, Milano, Italy
* Correspondence: giulia.lunghi@unimi.it

β-glucocerebrosidase (GCase) is a lysosomal glycohydrolase encoded by GBA gene, responsible for the catabolism of the sphingolipid glucosylceramide (GlcCer). Deficiency of this enzyme causes the lysosomal accumulation of GlcCer, leading to the onset of GCase-related pathologies, characterized by neurological impairment and neurodegeneration, which comprise Gaucher disease (GD) and GBA-dependent Parkinson’s disease (GBA-PD). Nevertheless, the relation between GCase loss of function and neurodegeneration is not understood so far. To dissect the possible molecular mechanism linking GCase deficiency and the consequent GlcCer accumulation with the onset of neuronal damage occurring in GCase-related pathologies, we developed an in-vitro human model of the neuronal form of GD represented by hiPSCs-derived dopaminergic neurons obtained from healthy subjects’ fibroblasts treated with 500 μM conduritol B epoxide (CBE), a specific GCase inhibitor. CBE-treated neurons present a progressive and time-dependent accumulation of GlcCer. Moreover, they recapitulate the neurodegenerative phenotype of GCase-related pathologies, presenting a significantly decreased expression of neuronal markers, such as Tau, MAP2, Neurofilament H, and PSD95. We also observed that GCase deficiency causes an enhanced lysosomal biogenesis and exocytosis, which leads to the extracellular release of uncatabolized GlcCer and to its accumulation also at the plasma membrane (PM) level. Interestingly, at the PM level GlcCer accumulates in specific signalling microdomains called “lipid rafts,” altering their sphingolipid pattern and protein content. In particular, we identified a reduction of complex gangliosides together with an enrichment of the active form of the non-receptor tyrosine-kinase c-Src. These data led us to speculate about the existence of a lysosome-PM axis responsible for the alteration of the PM architecture that can lead to the neuronal damage occurring in GCase-related pathologies.

## The Repositioning of the Antibiotic Moxifloxacin as a Novel Approach for Spinal Muscular Atrophy Treatment: Therapeutic Effects in SMNΔ7 Mice

Giovanna Menduti ^1,^*, Piotr Konieczny ^2^, Camille Januel ^3^, Cecile Martinat ^3^, Ruben Artero ^2^ and Marina Boido ^1^

^1^ Department of Neuroscience “Rita Levi Montalcini,” Neuroscience Institute Cavalieri Ottolenghi, University of Turin, Turin, Italy
^2^ Interdisciplinary Research Structure for Biotechnology and Biomedicine (ERI BIOTECMED), University of Valencia, Valencia, Spain
^3^ INSERM/UEPS UMR 861, Paris Saclay Université, I-STEM, 91100, Corbeil-Essonnes, France
* Correspondence: giovanna.menduti@unito.it

Spinal muscular atrophy (SMA) is a neurodegenerative disease affecting children, characterized by motor neuron (MN) impairment, skeletal muscle atrophy, and premature death. SMA is due to the mutation/deletion of the Survival Motor Neuron 1 (*SMN1*) gene; even if spared in case of disease, its human-specific copy (*SMN2* gene) fails in rescuing SMA phenotype since it produces a low amount of functional SMN protein. Improving therapeutic strategies aimed at increasing *SMN2* function is still a hot topic in the SMA field. Recently, by performing a screening of FDA-approved drugs, the antibiotic Moxifloxacin demonstrated to exert positive effects on *SMN2* exon 7 splicing, increasing SMN protein level on several SMA models (a Drosophila-based reporter system and SMA patients-fibroblasts). Here, we tested the effects of the Moxifloxacin administration in SMAΔ7 mice (a SMA type II murine model). The drug was daily injected into mice subcutaneously, from postnatal day 2 (P2) to P12. Weight assessment and behavioural and molecular analysis, respectively, showed a significant increase in body weight, motor skills, and SMN protein levels in different tissues (≥34% in the spinal cord, ≥91% in the quadriceps) in treated mice compared to the untreated ones. In addition, stereological and immunohistochemical analyses performed on lumbar spinal cord sections showed a delay in MN degeneration and a significant reduction in the levels of the apoptotic marker cleaved caspase 3 (≤46%) in treated mice. Moreover, concerning effects on neuroinflammation, astrogliosis (GFAP signal) was significantly reduced (≤48%) and a different degree of microglia ramification/activation was morphologically assessed in treated mice. Finally, histological analysis of skeletal muscles showed a significant increase in fibers area and Feret’s diameter in treated mice in comparison with untreated pups. Overall, the results demonstrated that Moxifloxacin can be potentially repositioned for the SMA treatment.

## Age-Dependent BBB Damage Favours Brain Iron Deposits, Activation of the Hepc/Fpn1 Pathway and Astrocytic-Neuronal Crosstalk

Mariarosa Mezzanotte ^1,^*, Giorgia Ammirata ^2^, Marina Boido ^3^, Serena Stanga ^3^ and Antonella Roetto ^1^

^1^ Translational Research Center for Innovative Medicine (TRECIM), Department of Clinical and Biological Sciences, University of Turin, Torino, Italy
^2^ Department of Clinical and Biological Sciences, University of Turin, Torino, Italy
^3^ Department of Neuroscience Rita Levi Montalcini, Neuroscience Institute Cavalieri Ottolenghi, University of Turin, Torino, Italy
* Correspondence: mariarosa.mezzanotte@unito.it

During aging, iron levels increase in the brain and accumulate in regions that are vulnerable to age-dependent neurodegeneration: the cerebral cortex and the hippocampus. However, the mechanism of iron regulation in the brain remains scarce. Here, we demonstrated for the first time the involvement of the Hepcidin/Ferroportin1 (Hepc/Fpn1) pathway in the metabolism of iron in the brain. We measured a remarkable reduction of Zonula occludens1 (ZO-1), a tight junction protein of the blood-brain barrier (BBB), indicating an increased permeability to iron in old mice; the alteration of iron homeostasis and its deposition in the brain drives neuroinflammation and oxidative stress. We found that Hepc is upregulated by the increase of iron content, and it acts as inhibitor of the iron exporter Fpn1. Interestingly, both in the cerebral cortex and hippocampus, Fpn1 colocalize specifically with astrocytes, while the iron storage protein ferritin light-chain colocalize with neurons. This differential distribution within neuronal tissue suggests that astrocytes drive iron shuttling in the brain and that neurons are unable to metabolize it. Moreover, we observed an increase of the ferritinophagy inductor Nuclear Receptor Coactivator 4 (NCOA4), which selectively degradates the ferritin heavy-chain (Ft-H) protein, in turn promoting the increase of the ferritin light chain (Ft-L) heteropolymers, which are more effective for iron storage. Altogether, these data highlight for the first time the involvement of the Hepc/Fpn1 axis and NCOA4 in brain iron increase during mice aging as a response to a higher iron flux in the central nervous system consequent to a BBB alteration.

## *C. elegans* Helps to Decipher the Mechanism Underlying Tau Toxicity in Tauophaties

Carmina Natale ^1,^*, Maria Monica Barzago ^1^, Margherita Romeo ^1^, Gloria Vegliante ^2^, Luca Colnaghi ^1^, Franca Orsini ^2^, Luana Fioriti ^2^, Roberto Chiesa ^2^, Elisa R. Zanier ^2^ and Luisa Diomede ^1^

^1^ Istituto di Ricerche Farmacologiche Mario Negri-IRCCS, Biochimica e Farmacologia Molecolare, Milano, Italy
^2^ Istituto di Ricerche Farmacologiche Mario Negri-IRCCS, Neuroscienze, Milano, Italy
* Correspondence: carmina.natale@marionegri.it

Abnormal tau phosphorylation and aggregation into bundles of filaments in the central nervous system is a common feature of a heterogeneous group of pathologies called tauopathies. Similarly to other misfolded proteins, tau oligomers more than fibrillar assemblies have been suggested to be the main responsible of toxicity. Hyperphosphorylated tau is able to spread in the brain, exerting its toxic function through a non-cell-autonomous mechanism. With the hypothesis that abnormal tau conformers play a causal role in driving toxicity, we conceived an original, integrated approach involving the use of recombinant human wild-type (WT) tau or tau carrying P301L mutation, cells overexpressing tau P301L, brain homogenates from WT or transgenic mice overexpressing tau P301L, and *C. elegans*. Cerebral homogenates from chronic traumatic brain-injured (TBI) mice showing widespread tau pathology were also employed. We found that recombinant tau oligomers but not monomers induced functional deficits in *C. elegans* consisting of neuromuscular impairment and altered synaptic transmission. Results were similar when worms were exposed to brain homogenates from P301L or TBI mice. Harsh protease digestion to eliminate the protein component of the brain homogenates from TBI or P301L mice, preincubation with anti-tau antibodies, or tau depletion by immunoprecipitation abolished the toxicity, indicating a pivotal role of abnormal tau conformers. These findings indicate that *C. elegans* represents a tractable model to investigate in vivo the toxicity of misfolded/aggregated tau, assessing its impact on neuromuscular function. This *C. elegans*-based platform can be successfully employed to test the ability of anti-tau compounds to interfere with the consequences of tauopathies.

## In-Vivo Targeting GPR17 by Montelukast Affects Survival and Disease Progression in a Gender-Dependent Manner in SOD1G93A Mice

T.P. Nhung Nguyen ^1,^*, Tiziana Bonifacino ^1^, Stefano Raffaele ^2^, Marta Fumagalli ^2^, Maria Pia Abbracchio ^3^ and Giambattista Bonanno ^1^

^1^ Department of Pharmacy, University of Genoa, Genoa, Italy
^2^ Department of Pharmacological and Biomolecular Sciences, University of Milan, Milan, Italy
^3^ Department of Pharmaceutical Sciences, University of Milan, Milan, Italy
* Correspondence: nguyen@difar.unige.it

Amyotrophic lateral sclerosis (ALS) is a multifactorial neurodegenerative disease leading to motor neurons death. Among the different pathological mechanisms, oligodendrocyte degeneration (OL) and OL precursor cell (OPC) maturation dysfunction contribute to ALS, guiding to myelin deterioration. An important regulator of OPC differentiation and survival is GPR17. However, at a specific stage, GPR17 needs to be downregulated for OPC maturation completion. We have previously shown that GPR17 expression is abnormally increased in the lumbar spinal cord (SC) of SOD1G93A mice and in SC-derived OPCs. Of note, the market available drug montelukast (MTK), a GPR17 antagonist, successfully restored the differentiation defects in primary OPC cultures from SOD1G93A mice. Here, we investigated the in-vivo effects of the MTK treatment in SOD1G93A mice. Two different MTK doses were used (10 or 30 mg/kg/24 h), starting at the early symptomatic phase of the disease (90 days of life). Survival probability was determined by the Kaplan–Meier analysis. Behavioural tests were performed three times per week to examine the progression of motor coordination (rotarod, beam balance tests); motor skills (gait, extension reflex tests); and muscle strength (paw-grip endurance, grip strength meter tests). The low MTK dose neither increased survival probability nor ameliorated disease progression. Accordingly, immunohistochemical analyses in lumbar SC did not highlight MTK-induced modifications of OL differentiation, myelin integrity, or neuroinflammatory readouts. On the contrary, the high MTK dose positively affected survival probability and strongly delayed weight loss in SOD1G93A female mice. Significant amelioration was also registered in behavioural tests. In contrast, SOD1G93A males were not significantly affected by MTK treatment. Our results suggest that in-vivo blocking GPR17 by MTK positively affects ALS progression in a gender-specific way and identify GPR17 as a novel pharmacological target.

## Optimization of AAV9 Gene Therapy for Spinal Muscular Atrophy with Respiratory Distress Type 1 Using In-Vivo Disease Model

Elisa Pagliari ^1,^*, Alessia Anastasia ^2^, Manuela Garbellini ^2^, Paola Rinchetti ^1^, Valentina Melzi ^2^, Michela Taiana ^1^, Giacomo Pietro Comi ^1^, Stefania Corti ^1^ and Monica Nizzardo ^2^

^1^ Department of Pathophysiology and Transplantation, Università degli Studi di Milano, Milan, Italy
^2^ Neurology Unit, Fondazione IRCCS Ca’ Granda Ospedale Maggiore Policlinico, Milan, Italy
* Correspondence: elisa.pagliari@gmail.com

Spinal muscular atrophy with respiratory distress type 1 (SMARD1) is a rare autosomal recessive motoneuron disease with infantile onset. It is caused by mutations in the *immunoglobulin mu-binding protein 2* (*IGHMBP2*) gene, which lead to a deficient amount of the encoded protein. The main clinical symptoms are distal muscular atrophy and diaphragmatic palsy, which requires supportive ventilation. Currently, there are no effective therapies available but only palliative treatments and only little research on this regard. Recently, adeno-associated virus 9 (AAV9)-mediated gene therapy showed promising results in preclinical models. To refine this approach, we compared the efficiency of two AAV9-*IGHMBP2* vectors, carrying different promoters, by administering them intracerebroventricularly (ICV) in SMARD1 mice model (*nmd*) during the presymptomatic phase of the disease at postnatal day 1. Expression analysis demonstrated a significant increase in the IGHMBP2 protein expression level compared to *nmd* mice. Treatments resulted in an extended survival time, higher body weight, and improvement of the motor behaviours. Histopathological analysis on mice muscles showed an increased innervation of the neuromuscular junctions and a recovery of fibers’ diameter; in addition, on spinal cords, we also observed an increased number of motoneurons associated to a reduced astrocyte gliosis. To support the translatability of the therapy, we confirmed the lack of a significative alteration of the toxicity biomarkers after the treatment, especially of the hepatic enzymes, usually. These data confirmed the efficacy of local administered gene therapy with a lack of relevant toxic effects. Although further investigations are needed on the possibility to expand the therapeutic window, up to now, these results provided a promising starting point for future application in clinical practice, paving the way for the development of an effective treatment for SMARD1 patients.

## Exosomes Derived from CAR-T Cells as Novel Treatment for Paediatric Brain Tumours

Marta Ibáñez Navarro ^1,^*, Susana García-Silva ^2^, Cristina Ferreras ^3^, Javier Saceda ^4^, Diego Plaza ^5^, Héctor Peinado ^2^, Antonio Pérez Martínez ^5^ and Lucía Fernández ^1^

^1^ Spanish National Cancer Research Centre (CNIO), Clinical Research Department, Haematological Malignancies, Madrid, Spain
^2^ Spanish National Cancer Research Centre (CNIO), Molecular Oncology Department, Tumor Microenvironment and Metastasis, Madrid, Spain
^3^ Hospital La Paz Institute for Health Research, IdiPAZ, Translational Research in Paediatric Oncology, Haematopoietic Transplantation, and Cell Therapy, Madrid, Spain
^4^ Department of Paediatric Neurosurgery, University Hospital La Paz, Madrid, Spain
^5^ Paediatric Haemato-Oncology Department, University Hospital La Paz, Madrid, Spain
* Correspondence: mibanez@cnio.es

Paediatric central nervous system (CNS) malignant tumors are the main cause of cancer-related death in children. Although current treatments have resulted in prolonged free survival rates (60–70%), the effects of surgery and chemo or radiotherapy in the developing brain of children may cause long-life neurologic irreversible effects, underlying the urgent need to find more specific and less toxic treatments. Chimeric Antigen Receptor T cell (CAR T)s have demonstrated potent anticancer efficacy in B-cell malignances, but in solid tumors, their efficacy is limited. The main hurdles for a successful CAR T cell therapy for paediatric brain tumors are lack of specific Tumor-Associated Antigens (TAAs), the hostile Tumor Microenvironment (TME), the difficulty to trespass the blood-brain barrier (BBB), and the need to avoid inflammation and neurotoxicity. NKG2D CAR T cells target up to eight different NKG2D ligands that are upregulated in CNS tumors and in Myeloid-Derived Suppressor Cells (MDSCs) and could overcome tumor heterogeneity and hostile TME. Additionally, they have shown efficacy against paediatric CNS tumor cell lines in vitro. Exosomes derived from CAR-T cells (exo-CAR T) maintain the TAA recognition ability and anti-tumor properties of their parental CAR T cells while presenting some advantages, including (1) their nanoscale size (30–120 nm), which can potentially facilitate trespassing of the BBB; (2) the lack of expression of inhibitory molecules, such as PD1, providing enhanced resistance to the immunosuppressive TME; and (3) inability to release inflammatory cytokines and thus minimizing the risk of Cytokine Release Syndrome. Thus, our project aimed to investigate the potential of exosomes derived from NKG2D CAR T cells (Exo-NKG2D CAR) as an advantageous therapeutic approach to treat paediatric CNS tumors. In this study, we aimed to isolate, characterise, and test the anti-CNS tumor ability of Exo-NKG2D CAR both in vitro and in a stereotaxic mouse model.

## Human Umbilical Cord-Derived Mesenchymal Stem Cells Limit Oligo-Dendrocytes Precursors Cells Damage in an In-Vitro Model of Encephalopathy of Prematurity

Marta Tiffany Lombardo *, Marta Lombardi and Claudia Verderio

Consiglio Nazionale Delle Ricerche (CNR), Istituto di Neuroscienze, Milano, Italy
* Correspondence: martatiffany.lombardo@in.cnr.it

To date, 1 in 10 babies is born premature, and even though the mortality decreased by 50% in the past years, brain damage still occurs, leading to motor, cognitive, and neuropsychiatric disabilities. The most frequent cause of these lesions is diffuse White Matter Insult, caused by impaired oligodendrocytes maturation and development of the myelin sheath with consequent altered cortical development. Due to their fundamental role in myelination and brain development during the third trimester of gestation, Oligodendrocyte Precursor Cells (OPCs) are considered the key cellular target in preterm brain injury. The aim of this study is to validate, in a simplified in-vitro model, human Umbilical Cord-Mesenchymal Stem Cells (hUC-MSCs) secretome as a strategy to limit the OPCs inflammatory damage caused by encephalopathy of prematurity. We investigated the effect of hUC-MSCs on survival, proliferation, and differentiation of OPCs, using a non-contact co-culture system. OPCs were obtained from five-day-old pup mice, selected and cultured in proliferating medium for 72 h followed by 48 h in differentiating medium with/without hUC-MSCs under control or inflammatory condition (medium conditioned by inflammatory microglia). MSCs benefit from being cultured in hypoxia (1% O_2_) compared to normoxia (21% O_2_). Therefore, hUC-MSCs were maintained in naïve and hypoxia conditions before being co-cultured with OPCs. At the end of the co-culture, OPCs were exposed to EdU or stained for MBP, OLIG2, and DAPI Ab for ICC analysis. hUC-MSCs promoted OPCs survival and differentiation both in control and under inflammatory insult, with an enhanced effect in the hypoxia-treated condition. These promising results indicate that hUC-MSCs secretome have protective effect on insulted OPCs, and further experiments are required to unravel the molecular pathways that drive this rescue.

This work was funded by H2020 PREMSTEM project.

## Unravelling Novel Players in Astrocyte-Mediated Phagocytosis

Veronica Giusti ^1,^*, Elena Giusto ^2^, Michele Sandre ^3^, Ludovica Iovino ^1^, Giuseppina Covello ^1^, Marta Giacomello ^1^, Luigi Bubacco ^1^, Elisa Greggio ^1^ and Laura Civiero ^4^

^1^ Department of Biology, University of Padova, Padova, Italy
^2^ IRCCS, San Camillo Hospital, Venice, Italy
^3^ Department of Neuroscience, University of Padova, Padova, Italy
^4^ Department of Biology/San Camillo Hospital, University of Padova/IRCCS, Padova, Italy
* Correspondence: veronica.giusti@phd.unipd.it

Elimination of unwanted and potentially harmful material is crucial for Central Nervous System development and function. Astrocytes are responsible for the clearance of dead cells, tissue debris, amyloidogenic-toxic proteins, and obsolete synapses. However, the molecular machinery involved in the recognition and degradation of such material is poorly characterized. Recent studies revealed that astrocytes contribute to synaptic clearance both in health and disease. The aim of this research is to dissect novel players of synapses clearance mediated by astrocytes following an unbiased approach. To achieve this goal, we optimized an in-vitro phagocytosis assay to evaluate the phagocytic capacity of astrocytes. In this assay, we used brain-purified neuronal terminals named synaptosomes, which manifest several features of live synapses, including the ability to be recognized and internalized by glial cells. To follow internalization in astrocytes, we conjugated synaptosomes with a pH-sensitive dye, which starts to emit bright red fluorescence within acidic organelles. Primary striatal astrocytes plated in multiple well plates were transfected with siRNA mouse library containing around 3000 hits, all potentially druggable. Fluorescence was acquired over time using high-content imaging system. We are now applying bioinformatic tools to prioritize hits for subsequent validation. Overall, this research highlights novel proteins involved in astrocytes-mediated phagocytosis that can be easily targeted in pathologies.

## Hyperactivity of Rac1 GTPase Pathway Affects the Development of Cortical Inhibitory Neurons

Carla Liaci *, Mattia Camera, Valentina Zamboni and Giorgio Roberto Merlo

Dipartimento di Biotecnologie Molecolari e Scienze per la Salute, Università di Torino, Torino, Italy
* Correspondence: carla.liaci@unito.it

GTPases of the Rho family are components of signaling pathways linking extracellular signals to the control of cytoskeleton dynamics. Among these, Rac1 plays key roles during brain development, ranging from neuronal migration to neuritogenesis, synaptogenesis, and plasticity. Rac1 activity is positively and negatively controlled by GEFs and GAPs respectively, but the specific role of each regulator in vivo is poorly known. The GTPase-activating protein ArhGAP15 is a specific negative regulator of Rac1, expressed during development in migrating cortical interneurons (CINs) and in a fraction of most subtypes of adult CINs. During development, loss of ArhGAP15 causes reduced morphological complexity and altered directionality of the leading edge of tangentially migrating CINs. Likewise, time-lapse imaging of embryonic CINs reveals a poorly coordinated directional control also during radial migration, possibly due to a hyperexploratory behavior. In the adult ArhGAP15^−/−^ cortex, the observed migration defects lead to subtle layering defects of distinct CIN subtypes, hyperexcitability of pyramidal neurons, spontaneous sub-clinical seizures, and increased susceptibility to the proepileptic drug pilocarpine. These results indicate that ArhGAP15 imposes a fine negative regulation on Rac1 that is required for normal control of directionality during CIN migration, with consequences on their laminar distribution and inhibitory function.

## Neural Stem Cell-Derived Exosomes Counteract Insulin Resistance-Induced Impairment of Brain Plasticity

Francesca Natale ^1,^*, Lucia Leone ^1^, Matteo Spinelli ^2^, Marco Rinaudo ^2^, Raimondo Sollazzo ^2^, Francesco La Greca ^2^, Saviana Antonella Barbati ^2^, Salvatore Fusco ^1^ and Claudio Grassi ^1^

^1^ Department of Neuroscience, Università Cattolica del Sacro Cuore—Fondazione Policlinico Universitario A. Gemelli IRCCS, Rome, Italy
^2^ Neuroscience, Università Cattolica del Sacro Cuore, Rome, Italy
* Correspondence: francesca.natale1@unicatt.it

Overnutrition and metabolic disorders induce cognitive deficits by affecting both neural stem cell (NSCs) niche and mature neurons. In particular, type 2 diabetes-related cognitive impairment is correlated with decreased adult neurogenesis in the hippocampus due to defective proliferation, differentiation, and cell survival. In physiological conditions, adult neural stem cells release extracellular vesicles (exo-NSC) contributing to intercellular communication and potentially regulating brain cell activity. Recently, numerous studies revealed the capability of exo-NSC to ameliorate brain functions and counteract cognitive decline occurring in experimental models of neurological diseases, but the underlying molecular mechanisms are still poorly understood. We investigated the effects of intranasal administration of exo-NSC on brain plasticity in a well-established experimental model of brain insulin resistance (i.e., mice fed with a high-fat diet). Our data demonstrated that intranasal administration of exo-NSC was able to deliver the vesicles into the hippocampus of mice and to restore the HFD-dependent proliferation/senescence unbalance of neurogenic niche. Chronic administration of exo-NSC also prevented HFD-induced memory impairment. Interestingly, exo-NSC seem to differently modulate intracellular molecular cascades in mature neurons and NSCs. In particular, exo-NSC prevented the inhibition of BDNF/TrKB/CREB signaling in differentiated neurons, whereas they rescued IRS1/FoxOs-mediated transcription of pro-proliferative genes in NSCs. Collectively, our findings highlight the role of extracellular vesicle cargo in the regulation of brain plasticity and provide evidence of the potential therapeutic effect of these vesicles against metabolic disease-related cognitive deficits.

## Investigating Patterns of White Matter Disconnections in Gliomas: A Single-Subject, Tractography-Based Approach

Umberto Villani ^1,^*, Erica Silvestri ^2^, Manuela Moretto ^1^, Diego Cecchin ^3^, Corbetta Maurizio ^1^,and Alessandra Bertoldo ^2^

^1^ Padovaneuroscience Center, University of Padova, Padova, Italy
^2^ Department of Information Engineering, University of Padova, Padova, Italy
^3^ Nuclear Medicine Unit, Department of Medicine, University Hospital of Padova, Padova, Italy
* Correspondence: umberto.villani@gmail.com

Gliomas are infiltrative brain tumours originating from white matter (WM) cells, often associated with poor prognosis. Having a clearer depiction of the involvement of WM pathways could be of paramount importance to predict the progression of the disease. To this end, diffusion MRI (dMRI) offers unparalleled potential for the in-vivo dissection of the human brain. Tractography techniques allow the reconstruction of WM fibre pathways connecting different brain regions. Although rich in information, full tractograms are difficult to navigate in search for the impairment of WM bundles due to the pathology. To obviate this issue, inspired by past work on defining structural disconnections, we here introduce a method to conveniently visualize the alteration caused by the presence of tumor on brain tissues. Forty-eight glioma patients were scanned at the University Hospital of Padova. Tumour core was manually segmented by an expert neuroradiologist using FLAIR, T1w (pre/post contrast medium), and T2w scans. Based on a rich dMRI protocol, we performed multi-shell multi-tissue spherical deconvolution and reconstructed the tractograms (10-M streamlines) using a probabilistic, anatomically constrained algorithm. In each subject, for every streamline in the tractogram, we initially tested whether it crossed the tumoral lesion. We subsequently transformed the subset of streamlines for which this overlap was found (i.e., affected streamlines) into a visitation map, detailing in each voxel how many altered streamlines were passing through it. We labelled these maps direct Structural Disconnection maps (dSD) in contrast to indirect ones, which compute the visitation maps projecting the lesion mask on selected population tractography atlases. Our study supports the use of dSD maps, which use subject-specific tractograms and are thus less prone to biases due to modified brain morphology (e.g., due to mass effect) potentially altering the predictive ability of the WM disconnection information.

## Toward a New Tolerogenic Strategy: Could Dendritic Cells Educated through Exposure to Specialized Pro-Resolving Mediators Be Possible Therapeutic Agents in Neuroinflammation?

Giada Pessina ^1,^*, Marta Bottero ^2^, Fabrizio Loiacono ^2^, Valerio Chiurchiù ^3,4^, Nicole Kerlero de Rosbo ^1^, Antonio Uccelli ^1,2^ and Giovanni Ferrara ^2^

^1^ Department of Neurology, Rehabilitation, Ophthalmology, Genetics, Maternal and Child Health, University of Genoa, L.go P. Daneo, 3, 16132 Genoa, Italy
^2^ IRCCS Ospedale Policlinico San Martino, L.go R. Benzi, 10, 16132 Genoa, Italy
^3^ Institute of Translational Pharmacology, National Research Council, Via del Fosso del Cavaliere 100, 00133 Rome, Italy
^4^ Laboratory of Resolution of Neuroinflammation, European Center for Brain Research, IRCCS Santa Lucia Foundation, Via del Fosso di Fiorano 64, 00143 Rome, Italy
* Correspondence: giada9820@gmail.com

Dendritic cells (DCs) play a crucial role in the immune system activation and in the regulation of immunological tolerance. DCs are present within the central nervous system (CNS), in the choroidal plexuses, and in the perivascular spaces regulating the CNS immune surveillance and tolerance in both mouse and human. In experimental autoimmune encephalomyelitis (EAE), a pathology mediated by encephalitogenic T-cell activation induced by auto-reactive DCs, the loss of immunological tolerance is one of the main autoimmune pathological mechanisms. We propose to generate tolerogenic DCs using a novel approach whereby DCs are exposed to specialized pro-resolving mediators (SPMs), a novel class of bioactive lipids that play a fundamental role in the resolution of inflammation. SPMs act as immunoresolvents by reducing the infiltration and activation of pro-inflammatory leukocytes, such as neutrophils, monocytes/macrophages, and T lymphocytes, and by shifting their immune response into an anti-inflammatory phenotype as well as promoting tissue healing and regeneration. Accordingly, we hypothesized that DCs conditioned by exposure to SPMs could acquire a tolerogenic phenotype and could reduce the activation of T cells, thus ameliorating EAE. qPCR analysis showed that differentiation of DCs induced to mature with LPS-INFγ in the presence of SPMs imparts a tolerogenic phenotype, with down-regulation of pro-inflammatory markers and concomitant upregulation of tolerogenic markers. Flow cytometry analyses confirmed that SPMs induce the anti-inflammatory phenotype of mature DCs by upregulating the surface markers of tolerance as well as other anti-inflammatory. Moreover, LPS-INFγ-activated DCs differentiated in the presence of SPMs maintained the upregulation of those tolerogenic markers after 24 h and 48 h and displayed a cytokine profile commensurate with a tolerogenic phenotype. In addition, activated T cells co-cultured with DCs or stimulated with the supernatant of DCs generated in the presence of SPMs, produced low levels of pro-inflammatory cytokines INFγ and IL-17 and displayed a reduced mRNA expression of the relative transcription factors. Our preliminary data point to a novel role of SPMs in the induction of a tolerogenic phenotype for DCs.

## Modulation of Neuroendocrinal and Peripheral Immunological Biomarkers by Rehabilitation in Sarcopenic Subjects

Federica Piancone ^1,^*, Marina Saresella ^1^, Francesca La Rosa ^1^, Ivana Marventano ^1^, Rossella Miglioli ^2^, Fabio Trecate ^2^ and Mario Clerici ^1^

^1^ IRCCS Fondazione Don Carlo Gnocchi ONLUS, Laboratorio di Medicina Molecolare e Biotecnologie, Milano, Italy
^2^ IRCCS Fondazione Don Carlo Gnocchi ONLUS, U.O. Riabilitazione, Milano, Italy
* Correspondence: fpiancone@dongnocchi.it

Sarcopenia is an aged-related condition characterized by loss of muscle mass and function, whose risk factors include, among others, inflammation and a complex imbalance of the neuroendocrine system. No pharmacological agents have been FDA approved for the treatment of sarcopenia, and the management of this disease is primarily focused on physical therapy for muscle strengthening and gait training. Because the crosstalk between the neuroendocrine and immune system is modulated by rehabilitation, the aim of this study was to verify the efficacy of the rehabilitation in reducing inflammation in sarcopenic patients. Sixty sarcopenic patients undergoing a specifically designed rehabilitation program were enrolled in the study. At the time of recruitment (T0) and at the end of the end of the rehabilitation program (30 days; T1), patients underwent a comprehensive geriatric multidimensional evaluation that included lower extremity function evaluation with the Short Physical Performance Battery (SPPB), fall risk assessment evaluation (Tinetti score), and the evaluation of performance in activities of daily living (Barthel index) as well as the analysis of the plasmatic concentration of proinflammatory (IL-1β, TNFα, IL-6, and IL-18) and antiinflammatory cytokines (IL-10) and the quantification in serum of neurotransmitters noradrenalin, adrenalin, dopamine, and serotonin. Rehabilitation resulted in a significant improvement of physical and cognitive conditions. This was accompanied by significantly increased concentrations of IL-10 and noradrenalin (*p* = 0.02 and *p* = 0.016, respectively) that were positively correlated with the improvement in the scores of the Tinetti (*p* = 0.02) and of the (SPPB) tests (*p* = 0.004). IL-18 concentation was significantly reduced as well at T1 (*p* = 0.008), and this was negatively correlated with Barthel index (*p* = 0.0085) and SPPB (*p*= 0.05) test scores. Results herein show a correlation between the rehabilitation efficacy and the reduction of the inflammation and identify the peripheral immunological and neuroendocrine biomarkers, which are modulated by rehabilitation.

## Differential Regulation of Haematopoiesis and Regulatory T-Cell Generation in Mouse Models of Glioma

Erika Ricci ^1,^*, Maria Cristina Mariani ^1^, Paolo Malatesta ^1^, Irene Appolloni ^2^, Davide Ceresa ^1^, Federico Ivaldi ^3^, Tiziana Vigo ^1^, Nicole Kerlero De Rosbo ^3^ and Antonio Uccelli ^1^

^1^ IRCCS Hospital San Martino, Genoa, Italy
^2^ Experimental Biology Unit, Department of Experimental Medicine (DIMES), University of Genoa, Genoa, Italy
^3^ Department of Neurosciences, Ophthalmology, Genetics, Maternal and Child Health, University of Genoa, Genoa, Italy
* Correspondence: erika.ricci.1985@gmail.com

Glioblastoma (GBM), the most common and malignant of all primary brain tumors in adults, is notorious for its immunosuppressive features and for its resistance to immunotherapy. The immunogenicity of glioma is lost during tumor progression. Indeed, low-grade glioma displays abundance of infiltrating lymphocytes, while in high-grade gliomas, a shift of the immune infiltrate towards a pro-tumorigenic phenotype has been observed. Tumor strategies to evade the immune system include sequestration of T cells in the bone marrow (BM) and promotion of regulatory T-cell (Treg) activity. Neuro-inflammation is known to elicit the generation of sympathetic nervous system (SNS) signals that control hematopoiesis and lymphopoiesis in BM and thymus. We propose that the progressive reduction of neuro-inflammation during brain tumor progression may alter SNS transmission to BM and thymus, impacting generation and release of lymphocytes, finally contributing to the process of tumor immunoediting. To assess our hypothesis, we have exploited a platelet-derived growth factor-B (PDGF-B) overexpressing mouse model of glioma. These mice can either develop low-grade (LG) or high-grade (HG) tumors. We have performed a single-cell gene expression analysis of LG and HG gliomas to define changes in the inflammatory signature of these tumors. We used flow cytometric analysis to monitor the differentiation of hematopoietic stem cells (HSC) in BM, of T-cell maturation and Treg generation in thymus, and of T and B lymphocytes in BM and blood. We observed an impairment of Treg generation in thymus of HG gliomas bearing mice, associated with a reduction of T and B lymphocytes in BM and blood and with promotion of HSC differentiation in BM. Our results clearly indicate that the maturation of immune cells in BM and thymus differs between LG and HG glioma-bearing mice. We are now assessing the possible signals which drive the regulation of immune cells that lead to promotion of LG vs. HG tumors.

## Effects of MAGL Inhibitor on Striatal Synaptic Dysfunction in a Mouse Model of Multiple Sclerosis

Krizia Sanna ^1,^*, Alessandra Musella ^2^, Francesca Romana Rizzo ^1^, Livia Guadalupi ^1^, Valentina Vanni ^2^, Diego Fresegna ^2^, Antonietta Gentile ^2^, Francesca De Vito ^3^, Sara Balletta ^1^, Silvia Caioli ^3^, Ludovic Collin ^4^, Anto Pavlovic ^4^, Sven Schippling ^4^, Diego Centonze ^1^ and Georgia Mandolesi ^2^

^1^ Medicina dei Sistemi, Università Tor Vergata, Roma, Italy
^2^ Synaptic Immunopathology Lab, IRCCS San Raffaele Pisana, Roma, Italy
^3^ IRCCS Istituto Neurologico Mediterraneo (INM) Neuromed, Pozzilli (IS), Italy
^4^ Roche, F. Hoffmann-La Roche Ltd. Roche, Innovation Center Basel, Basel, Switzerland
* Correspondence: krizia.sanna@live.it

Multiple sclerosis (MS) is an inflammatory neurodegenerative disorder in which the neuronal compartment is affected since the early stages of the disease. Data from MS patients and the MS rodent model, experimental autoimmune encephalomyelitis (EAE), have underscored a harmful but potentially reversible inflammatory synaptopathy in several brain area and a significant alteration of the endocannabinoid system (ECS). Studies from the EAE model have shed a light on the biological effects of endocannabinoids (eCBs)—anandamide (AEA) and 2-arachidonoylglycerol (2AG) —and their receptors (CB1R, CB2R, and TRPV). Of note, recent evidence showed that the inhibition of monoacylglycerol lipase (MAGL), the key hydrolytic enzyme responsible for 2-AG inactivation, can exert a surprising beneficial effect on EAE disease, but the mechanism is still unclear. Here, we took advantage of a reversible MAGL inhibitor (MAGLi) to investigate for the first time its effects on motor disability, neuroinflammation, and synaptopathy in EAE mice. Our data clearly indicate beneficial effects of MAGLi treatment in both ex-vivo and in-vivo conditions in EAE mice. We observed that MAGLi treatment is able to induce a less severe disease course accompanied by an improvement in motor activity evaluated by the grip strength test at the onset of the disease. Electrophysiological recordings revealed a selective recovery of the spontaneous glutamatergic current frequency in the striatum of EAE mice in association with an effective enzymatic MAGL inhibition and increased 2AG levels. Moreover, we explored the inflammatory status of the striatum, and by immunohistochemistry, we observed a significant reduction of the microglia activation. Overall, we demonstrated that an up-regulation of the endocannabinoid tone induced by MAGL inhibition is potentially involved in the recovery of both inflammatory status and glutamatergic alterations mediated by CB1 receptor occupancy in EAE mice.

## Air Pollution and Neuroinflammation

Giulia Terribile * and Giulio Sancini

School of Medicine and Surgery, University of Milano-Bicocca, Monza, Italy
* Correspondence: g.terribile1@campus.unimib.it

Air pollution consists of a complex mixture of chemicals, particular matter (PM), organic compounds, metals, ions, and elemental carbon, which can harm living organisms, including humans. The several PM adverse effects may relate to its physicochemical characteristics, including mass, size, number, surface area, concentration, source, and composition. Since the last decade, the central nervous system (CNS) has also been proposed to be a target organ for the detrimental effects of airborne pollutants. Emerging evidence from epidemiological, clinical, and experimental studies suggest that certain neurological diseases, such as Alzheimer’s disease, may be strongly associated with ambient air pollution. Although the precise mechanisms underlying neurodegenerative diseases still remain elusive and are not fully understood, environmental pollution is believed to exert its neurotoxic function through oxidative stress, glial activation, and cerebrovascular damage. The aim of our study is to explore the effects of airborne pollution on CNS elements and brain homeostasis focusing on microglia, brain cells of nonneural origin, as predominant regulators of neuroinflammation. To explore the effects of airborne pollution on microglia we treated, at different timepoints, an immortalized line of murine microglia (i.e., BV2) with different concentration of a standard reference material of diesel exhaust particles (DEP), one of the main component of airborne pollutants at urban area. After the treatment, we evaluated cell viability, cell morphology, and intracellular calcium waves by means of calcium imaging technique. Our preliminary data suggest that BV2 cells actively interact with DEP after 24 h treatment. DEP seems to be also chemoattractant factor for BV2, but the exact mechanisms are still unknown. We are going to deeply investigate how DEP is able to interact with BV2 functions, thus triggering neuroinflammation and neurodegeneration.

## Translocator Protein Modulation of Human Microglia Activity: The Involvement of Neurosteroids Production

Chiara Tremolanti *, Lorenzo Germelli, Barbara Costa and Eleonora Da Pozzo

Department of Pharmacy, University of Pisa, Pisa, Italy
* Correspondence: chiara.tremolanti@phd.unipi.it

Dysregulation of microglial activity is related to the development of chronic neuroinflammation and neurodegenerative diseases. The mitochondrial translocator protein (TSPO, 18 kDa) has emerged as a promising target against neuroinflammation due to its overexpression in activated glia. Several cellular processes have been proposed to underlie TSPO immunomodulatory activity, including neurosteroidogenesis induction. However, the precise role of TSPO in the modulation of reactive microglia is still unclear. We investigated the potential role of TSPO during the inflammatory response in a model of immortalized human inflamed microglia. In addition, we aimed to elucidate the mechanism underlying its activity. Our results demonstrated that TSPO stimulation by the use of a selective ligand attenuated microglia activation promoting the shift towards the restorative phenotype. Moreover, this phenomenon was abolished in the presence of an inhibitor of the neurosteroidogenesis cascade, suggesting a possible role for neurosteroids in the modulation of microglial phenotype. Therefore, we investigated the putative neurosteroidogenic activity of microglia, and our results showed that microglia express all the key members for neurosteroid production, including TSPO, StAR, and CYP11A1, which convert cholesterol into pregnenolone as the first limiting step of neurosteroids synthesis. In line with these results, pregnenolone was accumulated by human microglia cells in a time-dependent manner, and the pharmacological stimulation of TSPO drastically increased pregnenolone release. In conclusion, the results obtained in our experimental setting suggested that TSPO contributes to the preservation of microglia well-being, exerting a negative regulation on neuroinflammatory mechanisms, and this activity could be attributed to the induction of neurosteroidogenesis.

## The Modulation of Mitochondria Function as a Possible Mechanism for GM1 Oligosaccharide-Derived Neuroprotection

Maria Fazzari ^1,^*, Giulia Lunghi ^1^, Erika Di Biase ^1^, Matteo Audano ^2^, Pamela Fato ^1^, Laura Mauri ^1^, Maria Grazia Ciampa ^1^, Gabriella Tedeschi ^3^, Nico Mitro ^2^, Sandro Sonnino ^1^ and Elena Chiricozzi ^1^

^1^ Department of Medical Biotechnology and Translational Medicine, University of Milano, Segrate, Italy
^2^ Department of Pharmacological and Biomolecular Sciences, University of MIlano, Milano, Italy
^3^ Department of Veterinary Medicine, University of Milano, Milano, Italy
* Correspondence: maria.fazzari@unimi.it

Parkinson’s disease (PD) is a neurodegenerative disorder characterized by the progressive loss of dopaminergic (DA) neurons in the brain substrantianigra. Although its etiopathogenesis is still poorly understood, the mitochondrial (mit) dysfunction was described to have a crucial role in the exacerbation of neuronal degeneration. Hence, mit-targeted protective compounds capable of minimizing mit dysfunction constitute hopeful therapeutic strategies for PD. Here, we describe the properties of GM1 oligosaccharide (OligoGM1), the bioactive portion of GM1 ganglioside, that, by interacting with and activating the NGF TrkA receptor at the cell surface, triggers crucial cellular pathways responsible for mit neuroprotection. Using proteomic and biochemical approaches, we demonstrated that OligoGM1 is able to induce the mitochondriogenesis and to enhance the mit function. Wild-type Neuro2a cells treated with OligoGM1 showed an increased number of mitochondria and, at functional level, an increased expression of mit complexes, boosted ATP levels, and mit respiration. Importantly, OligoGM1 treatment determined the rescue of mit activity and respiration in a Neuro2a model of mit dysfunction. On the other hand, OligoGM1 proved to efficiently counteract both in vitro and in vivo the neurotoxicity associated to 1-methyl-4-phenyl-1,2,3,6-tetrahydropyridine (MPTP), a PD-linked neurotoxin that acts by inhibiting the mit complex I. Specifically, OligoGM1 pre-treatment strongly reduced the mit ROS overproduction and P38 MAPK hyper-phosphorylation due to MPTP exposure leading to increased cell viability and neurite network in Neuro2a cells and DA neurons together with enhanced ATP levels and mit complexes expression. Collectively, our data indicate that OligoGM1 is able to protect neurons possibly via mit function restoration and oxidative stress reduction, opening a new perspective for the use of OligoGM1 in diseases where these organelles are compromised, including PD.

## Stem Cells Differentiation in a 3D Environment for the Study of ALS

Eveljn Scarian ^1,^*, Matteo Bordoni ^2^, Riccardo Cotta Ramusino ^3^, Valentina Fantini ^1^, Stephana Carelli ^4^, Orietta Pansarasa ^5^ and Cristina Cereda ^5^

^1^ Department of Brain and Behavioral Sciences, University of Pavia, Pavia, Italy
^2^ Centro di Eccellenza sulle Malattie Neurodegenerative, Dipartimento di Scienze Farmacologiche e Biomolecolari (DiSFeB), Università degli Studi di Milano, Milano, Italy
^3^ Department of Biology and Biotechnology “L. Spallanzani,” University of Pavia, Pavia, Italy
^4^ Department of Biomedical and Clinical Sciences “L. Sacco,” University of Milan, Milano, Italy
^5^ Genomic and Post-Genomic Unit, IRCCS Mondino Foundation, Pavia, Italy
* Correspondence: eveljn.scarian@mondino.it

Amyotrophic lateral sclerosis (ALS) is a neurodegenerative disease that affects both upper and lower motor neurons in cortex, brainstem, and spinal cord, causing weakness, muscle atrophy, and spasticity. Unfortunately, there are only symptomatic treatments available. An important innovation of recent years is 3D bioprinting, which allows the creation of a 3D model for the study of interaction between cells and between cells and environment. Moreover, an important newness is the use of induced pluripotent stem cells (iPSCs). They are pluripotent stem cells derived from adult somatic cells, which can allow the creation of a more realistic pathological model advancing the field of personalized medicine. The aim of this work was the development of a protocol of 3D stem cells differentiation and their characterization. We first obtained iPSCs from peripheral blood mononuclear cells (PBMCs) of ALS patients and healthy subjects and differentiated them in neural stem cells (NSCs). NSCs were then included in Cellink Bioink and printed in 3D structures. Cells were differentiated in 3D in motor neuron progenitors (MNPs), immature MNs, and at the end mature MNs. Every step was tested for cells viability and characterized by confocal microscopy and RT-qPCR. At the end, we tested the electrophysiological characteristics of included Mouse Motor Neuron-Like Hybrid Cells (NSC34). We found that included NSCs maintain a good proliferation rate during the differentiation in 3D. Moreover, we confirmed the good differentiation process both by confocal microscopy and RT-qPCR, using specific markers of the different steps. Finally, we confirmed that NSC34 cells maintain their electrophysiological characteristics when printed in a 3D structure. In conclusion, we confirmed that 3D bioprinting can be considered a good model for the study of the pathogenesis of ALS allowing the growth and proliferation of cells in a more physiological environment.

## Pathological Hallmarks in Brain Organoids Derived from sALS Patients

Matteo Bordoni ^1^, Eveljn Scarian ^2,^*, Emanuela Jacchetti ^3^, Valentina Fantini ^2^, Manuela Teresa Raimondi ^3^, Stephana Carelli ^4^, Orietta Pansarasa ^5^ and Cristina Cereda ^5^

^1^ Centrodi Eccellenza sulle Malattie Neurodegenerative, Dipartimentodi Scienze Farmacologichee Biomolecolari (DiSFeB), Università degli Studi di Milano, Milano, Italy
^2^ Department of Brain and Behavioral Sciences, University of Pavia, Pavia, Italy
^3^ Department of Chemistry, Materials and Chemical Engineering “Giulio Natta,” Politecnico di Milano, Milano, Italy
^4^ Department of Biomedical and Clinical Sciences “L. Sacco,” University of Milan, Milano, Italy
^5^ Genomic and Post-Genomic Unit, IRCCS Mondino Foundation, Pavia, Italy
* Correspondence: eveljn.scarian@mondino.it

Amyotrophic lateral sclerosis (ALS) is considered a non-cell autonomous disorder, and many cell types contribute to motor neurons death. The lack of effective treatments is probably due to the absence of a realistic model that can recapitulate early and late pathogenic mechanisms. Cerebral organoids are pluripotent stem cell-derived self-organizing structures that recapitulate brain development and allow in-vitro generation of 3D tissues. We developed a new method for the generation of motor neuron organoids that can be used for the study of pathogenic mechanisms in ALS. The aim of the work was to characterize a 3D organoid model for the study of ALS pathogenesis. We started from iPSCs obtained from healthy controls and sALS patients. We differentiated iPSCs into neural stem cells (NSCs). NSCs were dissociated using StemPro Accutase and a cell strainer. Then, NSCs were plated on low-attachment six-well plate and were cultured in floating conditions using an orbital shaker. NSCs were differentiated in these conditions into motor neuron progenitors (MNPs), immature motor neurons (MNs), and finally mature MNs. We then characterized cells by phase-contrast and confocal microscopy. We found that brain organoids derived from sALS patients were smaller and with irregular morphology compared to healthy controls. Using the GFAP marker, we found that sALS organoids have a thicker glial layer compared to healthy controls. We also found that healthy controls organoids show longer neurites compared to sALS organoids. Finally, we found a diverse composition of cell populations. Indeed, healthy controls organoids show a higher amount of differentiated cells compared to sALS organoids. In conclusion, brain organoids represent a promising tool for the investigation of pathogenic mechanisms of ALS. Indeed, we found typical pathological hallmarks of the pathology, such as the presence of gliosis, the smaller length of neurites, and the decreased level of mature MNs.

## Characterization of the Neuromuscular junction in iPSC-Derived 2D and 3D FUS-ALS Model Systems

Beatrice Silvestri ^1,^*, Alessandro Rosa ^1^, Maria Giovanna Garone ^1^ and Valeria De Turris ^2^

^1^ Dipartimento di Biologia e Biotecnologie “Charles Darwin,” Università di Roma “La Sapienza,” Roma, Italy
^2^ Istituto Italiano di Tecnologia, Roma, Italy
* Correspondence: beatrice.silvestri@uniroma1.it

Amyotrophic lateral sclerosis (ALS) is a neurodegenerative disease characterized by motor neurons (MNs) death in the spinal cord and brain, leading the loss of skeletal muscle mass (muscle atrophy). So far, it has been difficult to investigate the molecular pathways of ALS because of the lack of suitable cell model system. The use of induced pluripotent stem cells (iPSC) carrying ALS mutations introduced by gene editing represents a valuable opportunity for the study of this pathology. Such a cell model system has indeed provided important improvements on the understanding of the molecular processes involved in ALS pathology. Mutations in the FUS protein have been reported in several ALS patients. It has been shown that a hallmark of ALS disease is the abnormal cytoplasmatic accumulation of FUS-mutated protein. Preliminary data collected in our lab highlight an aberrant increase in axon branching and growth in MNs derived from FUS-mutated iPSCs. Moreover, an interesting aberrant crosstalk between FUS protein and the RNA-binding protein HuD, leading to an upregulation of HuD levels, has been identified. This interaction results in the consequent upregulation of some HuD targets, such as the axonal proteins GAP43 and NRN1. The principal aim of this project is to analyze the importance of GAP43 involvement in aberrant axon phenotypes and confirm whether this increase in neurite branching can lead to neuromuscular disruption. Notably, neuromuscular junction (NMJ) degeneration has been observed as an early pathogenic event in ALS. To recapitulate the NMJ circuit formation in vitro, we took advantage from iPSCs to obtain neural-muscle systems by 2D co-cultures and 3D neuromuscular organoids. The study of NMJ impairment will be conducted by morphological and functional assays to assess the contribution of FUS mutation in ALS progression. Finally, understanding of these cellular processes represents a crucial step for to the development of more personalized therapies.

## Extracellular Vesicles Showed Different Immune Phenotypes in Patients with Amyotrophic Lateral Sclerosis

Daisy Sproviero ^1,^*, Eleonora Corridori ^2^, Stella Gagliardi ^1^, Luca Diamanti ^3^, Matteo Bordoni ^2^, Orietta Pansarasa ^1^ and Cristina Cereda ^1^

^1^ Genomic and Post-Genomic Unit, IRCCS Fondazione Mondino, Pavia, Italy
^2^ Centro di Eccellenza sulle Malattie Neurodegenerative, Dipartimento di Scienze Farmacologiche e Biomolecolari, Università degli Studi di Milano, Milano, Italy
^3^ Neuro-Oncology Unit, IRCCS Fondazione Mondino, Pavia, Italy
* Correspondence: daisy.sproviero@mondino.it

Background. Extracellular vesicles (EVs) play central role in inflammatory processes, and they could be plausible targets in ALS related to immunological reaction to motor neuron loss. We previously demonstrated that leukocyte-derived EVs are upregulated in ALS patients, and they can be considered markers of disease progression. 

Objectives. The aim of this study was to investigate specific immunological surface markers on Large and Small EVs (LEVs and SEVs) from plasma of sporadic ALS (sALS) patients and healthy donors (CTRLs).

Methods. EVs were isolated from plasma of 50 sALS and 30 CTRLs by differential centrifugation and characterized by Nanoparticle Tracking Analysis (NTA), Atomic Force Microscopy (AFM), and COlorimetric NANoplasmonic method (CONAN). For a simultaneous identification of 37 surface protein markers in each sample, we used a multiplex bead-based flow cytometric assay (MACSPlex Exosome Kit).

Results. Endosome-specific tetraspanins (CD63 and CD9), leucocyte, platelet and endothelial cell adhesion molecules (leukocyte common antigen (CD45), platelet endothelial cell adhesion molecule (PECAM-1, CD31), human Integrin alpha 5 (CD49e), E-selectin (CD69), and B-lymphocyte antigen (CD20) were more expressed in SEVs derived from CTRLs than in sALS patients (*p* < 0.05). LEVs derived from CTRLs were enriched in platelet surface expression of CD62P (P-selectin) (*p* < 0.05) and CD63 (*p* < 0.001) compared to sALS patients.

Discussion. Endosome-specific tetraspanins decrease in both LEVs and SEVs in accordance with the autophagy-endolysosomal system dysregulation described in ALS. LEVs from sALS patients also had fewer platelet activation markers, in line with the literature, as suggested by the platelet variation in blood of ALS patients. These data suggest that LEVs and SEVs carry different surface markers which might discriminate their role in ALS pathogenesis.

## Exceptionally Potent Human Monoclonal Antibodies Are Effective for Prophylaxis and Therapy of Tetanus in Mice

Marika Tonellato ^1,^*, Marco Pirazzini ^1^, Alessandro Grinzato ^1^, Davide Corti ^2^, Sonia Barbieri ^2^, Oneda Leka ^1^, Francesca Vallese ^1^, Chiara Silacci-Fregni ^3^, Luca Piccoli ^3^, Eaazhisai Kandiah ^4^, Giampietro Schiavo ^5^, Giuseppe Zanotti ^1^, Antonio Lanzavecchia ^3^ and Cesare Montecucco ^1^

^1^ Department of Biomedical Sciences, University of Padova, Padova, Italy
^2^ Humabs BioMed SA, Vir Biotechnology, Bellinzona, Switzerland
^3^ Institute for Research in Biomedicine, Università della Svizzera Italiana, Bellinzona, Switzerland
^4^ European Synchrotron Radiation Facility, (ESRF), Grenoble, France
^5^ Department of Neuromuscular Diseases, Queen Square Institute of Neurology, UK Dementia Research Institute, University College London, London, UK
* Correspondence: marika.tonellato@gmail.com

Tetanus neurotoxin (TeNT) is the causative agent of tetanus, a life-threatening disease of vertebrates, including humans, characterized by neurogenic muscle rigidity and spasticity. Although tetanus can be prevented by a very effective vaccine, worldwide clinical practice in the emergency rooms is the administration of anti-TeNT immunoglobulins (TIG), which are used both for prophylaxis to avoid tetanus development in wounded patients or for therapy to treat patients already carrying tetanus symptoms. TIG is produced from the blood of hyperimmune individuals, either humans or horses (in developing countries). As such, it exposes patients to several possible side-effects, including infections by still-unknown pathogens as well as dangerous anaphylactic reactions. Human monoclonal antibodies (humAbs), which are emerging as superior therapeutics against several diseases, could overcome the drawbacks of TIG. Here, we screened the immortalized memory B cells pooled from the blood of immunized human donors and isolated two TeNT-specific humAbs, dubbed TT104 and TT110, that display an unprecedented neutralization ability against TeNT neurotoxicity. We determined the epitopes recognized by TT104 and TT110 via cryo-EM and defined how they interfere with the mechanism of neuron intoxication. These analyses pinpointed two novel mechanistic aspects of TeNT activity in neurons unraveling at the same time as the molecular bases of the exceptional neutralization ability of TT104 and TT110. Crucially, the combination of TT104 and TT110 displayed a prophylactic activity in mice when injected long before TeNT. Moreover, the two Fab derivatives neutralized TeNT in post-exposure experiments. In both these two paradigms of experimental tetanus, TT104 and TT110 humAbs and their Fabs derivates display an activity fully comparable to TIG. In conclusion, they meet all requirements for being considered for prophylaxis and therapy of human tetanus and are ready for clinical trials.

## Identification of the Transition Frequency from Theta to Alpha Band in Patients with Neurodegenerative Disease

Elisabetta Vallarino ^1,^*, Sara Sommariva ^1^, Dario Arnaldi ^2^, Flavio Nobili ^2^ and Michele Piana ^1^

^1^ Dipartimento di Matematica, Università degli Studi di Genova, Genova, Italy
^2^ IRCCSOspedale Policlinico San Martino, Dipartimento di Neuroscienze, Genova, Italy
* Correspondence: vallarino@dima.unige.it

It is well known that brain activity is modulated by oscillations within different frequency bands, each of them related to specific brain states. Common practice in electroencephalography (EEG) signal processing consists in analysing the data at specific frequency bands chosen according to the interest and target of the study. It is straightforward that an optimal identification of the frequency bands is the starting point to reach good results. Many studies still rely on a standard frequency band subdivision; however, a subject-specific frequency band subdivision is gaining more interest. This is of utmost importance, for instance, when dealing with data from Alzheimer’s patients since it has been demonstrated a progressive shift towards the low frequencies of the power spectrum profile with the progression of the disease.

The key point in a good subject-specific frequency band subdivision is the identification of the individual alpha peak (IAP) and the transition frequency (TF) from theta to alpha band. While the literature presents robust methods for the identification of IAP, there still is a lack of methods for the identification of the TF. The main method reported in literature requires both resting state and task EEG recordings; however, this is limiting, as it is not rare to only have resting state data at disposal for the analysis.

We propose a novel method for the identification of the TF, which only requires resting state EEG recordings. The key idea of the presented method is to identify two EEG channel groups, characterized by a strong presence of alpha activity in the first group and theta activity in the second. The two groups play the role of the resting state and task EEG recordings in the classical method. We validated the method on an EEG open-source dataset from both Parkinson’s patients and healthy subjects. The novel method that we propose is easy to implement and yet very robust; thus, it can be of great interest for the neuroscientific community.

## A Bidirectional Relationship between Alzheimer’s Disease and Sleep Fragmentation in 5xFAD Mice

Valeria Vasciaveo ^1,^*, Antonella Iadarola ^2^, Elena Tamagno ^1^, Alessandro Cicolin ^2^ and Michela Guglielmotto ^1^

^1^ Dipartimento di Neuroscienze, Neuroscience Institute Cavalieri Ottolenghi, Torino, Italy
^2^ Centro Medicina del Sonno, AOU Città Della Salute e della scienza, Molinette, Dipartimento di Neuroscienze, Torino, Italy
* Correspondence: valeria.vasciaveo@unito.it

Alzheimer’s disease (AD) is the most common age-related disorder, characterized by loss of memory and cognitive functions. It is due to both genetic and environmental factors, and it is correlated to aging. Recent studies have demonstrated that sleep disorders can be a risk factor for developing AD. Indeed, between AD and sleep disorders a bidirectional relationship exists. Actually, the acute effect of sleep disorders results in an increase in Ab concentration; besides, the sleep quality in AD patients is impaired, leading to a possible further accumulation of Ab. Literature data demonstrate a correlation between AD and acute sleep deprivation. In this study, we performed and validated a mouse model of AD and sleep fragmentation, a sleep disorder that mimics more correctly a real condition of intermittent and continuous awakening. The fragmentation method lasts 30 days, all day long, and includes a time-controlled tilt, which swings every 3 min for about 10-sec epochs. We analysed the effect of sleep fragmentation in 5xFAD and wild-type (wt) mice of two months of age since 5xFAD mice at this age already develop senile plaques. In this study, we observed in fragmented 5xFAD and wt mice respect to their control (no-fragmented) an increase in the amount of Ab1-42 production, tau phosphorylation, and GSH/GSSG ratio. Moreover, we observed a modulation in the expression of the kinases involved both in the oxidative stress pathway and in tau phosphorylation, such as pJNK and pERK in fragmented wt and 5xFAD mice. For these reasons, we examined in depth the modulation of tau protein in mice expressing human tau. In conclusion, since the first signs of AD appear 15–20 years after its pathogenesis begins, it is possible to say that a bad sleep quality could be a risk factor for AD but also an aggravating of the pathogenesis and probably speeding it up. A more in-depth study could lead to the development of new preventive therapies in patients with chronic sleep disorders.

## The Effect of Exosomes Isolated from Murine Adipose-Derived Stem Cells on Two Motor Neurons Disorders: ALS and SMA

Federica Virla ^1,^*, Sylwia Dabrowska ^1^, Ilaria Scambi ^1^, Molakun Bankole ^1^, Marina Boido ^2^ and Raffaella Mariotti ^1^

^1^ Department of Neurosciences, Biomedicine and Movement Sciences, University of Verona, Verona, Italy
^2^ Department of Neuroscience Rita Levi Montalcini, University of Turin, Turin, Italy
* Correspondence: federica.virla@univr.it

The use of stem cells represents a possible treatment for neurodegenerative disorders, like amyotrophic lateral sclerosis (ALS) and spinal muscular atrophy (SMA). In particular, their beneficial action seems to be due to the paracrine release of exosomes, main mediators of intercellular communication. Indeed, through the release of their content (proteins, miRNA and nucleic acids), they are able to promote neurogenesis, inhibit apoptosis, and enhance immunomodulation in different pathophysiological contexts, recapitulating the effect of origin cells.

Although ALS and SMA are two distinct neurodegenerative diseases caused by different pathogenic mechanisms, our aim is to investigate the protective influence of exosomes in two different in-vivo models. We therefore isolated and characterized exosomes from murine adipose-derived stem cells (ASC-exosomes) and delivered them via intranasal administration in the SOD1(G93A) mice, the murine model of ALS, and with intracerebroventricular injections in the SMNΔ7 murine model, the most widely used one for SMA.

The results showed that ASC-exosomes could improve the motor performance of animals, both in treated SOD1(G93A) and SMNΔ7 mice. They could also protect lumbar spinal cord motor neurons from neurodegeneration and modulate the neuroinflammation in the central nervous system. Moreover, in the peripheral tissues, we could observe a higher number of innervated neuromuscular junctions and an attenuated skeletal muscle atrophy in the treated SMNΔ7 group. These outcomes could allow to better understand the effects of ASC-exosomes and to target them as a possible approach in the treatment of neurodegenerative disorders.

## Effects of Traffic-Related Air Pollution on the Development of Alzheimer’s Disease: The Role of the Olfactory Pathway

E. Scarcello ^1^, T. Wahle ^1,^*, A. Sofranko ^1^, J. van Rüschen ^1^, I. Masson ^1^, C. Albrecht ^2^, G. Bredeck ^1^, M. Busch ^1^, P. Fokkens ^3^, J. Boere ^3^, F. R. Cassee ^3,4^ and R. P. F. Schins ^1^

^1^ Air Pollutants and Brain Aging Research Group, IUF-Leibniz Research Institute for Environmental Medicine, Duesseldorf, Germany
^2^ IUF-Leibniz Research Institute for Environmental Medicine, Duesseldorf, Germany
^3^ National Institute for Public Health and the Environment (RIVM), Bilthoven, The Netherlands
^4^ Institute for Risk Assessment Sciences, Utrecht University, Utrecht, The Netherlands
* Correspondence: eleonora.scarcello@gmail.com

**Abstract**: Ambient air pollution, a heterogenous mixture of gases and fine and ultrafine particles, has been shown to potentially cause adverse health effects, including respiratory and cardiovascular diseases. More recent investigations have also shown that ultrafine particles can deposit in the brain and increase the risk of developing neurological disorders, such as Alzheimer’s disease (AD). In previous investigations, we already showed that diesel engine exhaust can aggravate amyloid plaque formation and motor function impairment in a mouse AD model. However, not only the molecular and cellular mechanisms behind it are still unknown but also if there are any biomarkers indicating air pollution’s effects on the brain. In order to address these questions, we exposed female 5X Familial AD (5XFAD) mice and their female wild-type (WT) littermates for 5 h/day, 5 days/week during two or four consecutive weeks at a traffic-dominated location to concentrated ambient particles (CAPs), particle filtered air (FA), or clean air (CA). Our preliminary results revealed that traffic-related air pollution accelerates AD-like pathologies in the 5xFAD mice, albeit to a different extent by CAPs and FA exposures. Analysis of the olfactory bulbs of 5xFAD vs. WT mice from the respective exposure groups by cytokine array indicated that the olfactory route might be involved in these adverse effects. Potential individual biomarkers were thus chosen for further evaluation by immunohistochemical analysis, ELISA, or Western blot.

**Acknowledgments**: The work leading to these results has received funding from the European Union’s Horizon 2020 research and innovation programme under grant agreement No 814978 (TUBE) and a cross-border grant awarded by the Alzheimer Forschung Initiative (AFI, Germany) and Alzheimer Nederland.

## A Strange Case of Foetal Hydrops: The Mystery of the Vein of Galen

Roberta Pintus ^1,^*, Maria Antonietta Marcialis ^2^, Maria Cristina Pintus ^2^, Flaminia Bardanzellu ^1^, Viviana Marinelli ^2^ and Vassilios Fanos ^1^

^1^ Neonatal Intensive Care Unit, Department of Surgery, University of Cagliari, Cittadella Universitaria, SS 554, km 4.5, Monserrato, 09042 Cagliari, Italy
^2^ Neonatal Intensive Care Unit, University of Cagliari, Cittadella Universitaria, SS 554, km 4.5, Monserrato, 09042 Cagliari, Italy
* Correspondence: gomberta@icloud.com

We present a case report of a preterm neonate born at 31 weeks and 6 days of gestational age (birth weight 2300 g) by emergency c-section due to fetal hydrops, mainly characterized by abundant pleural fluid with lungs compression and mild pericardial effusion. Echocardiography showed a good biventricular function. At birth, the baby was asphyxiated (Apgar 3 and 4) and then intubated and underwent external cardiac massage, treated with a dose of endotracheal surfactant in the birth room. She was then transferred to the Neonatal Intensive Care Unit in extremely severe conditions. The postnatal echocardiography showed iso-systemic lung hypertension without structural cardiopathy. The newborn was treated with invasive ventilation for 13 days and for 11 with non-invasive ventilation. The karyotype, the haemoglobin, the metabolism, and the infection parameters were all normal. The brain sonography was normal up to the first month of life, when it showed multiple micro-calcifications in the posterior right frontal parenchyma. The following magnetic resonance examination showed a small ectasia of the straight sinus with an apparent aneurismatic dilation of the vein of Galen. It will be re-evaluated with angiography sequences.

The following brain sonographies evidenced with some difficulties the aneurismatic dilation of the vein of Galen, but they showed several doppler velocity grams inside the same vein. Two were arterial, characterized by an IR of 0.5 and one was venous. The pericallosal artery showed a systolic velocity that ascends in a steep way and one diastolic that showed a notch and an IR of 0.9.

The echocardiography is normal.

Now, the new-born is in discreet general conditions and is gaining weight.

At nine months, the Magnetic Resonance and the angio-magnetic resonance examination showed a minimal dimensional increment of the venous ampulla of Galen and the ventricular system. Nevertheless, the psycho-behavioural and motor profile of the baby was normal.

## Functional Assessment of KCNB1 Variants: Loss-Versus Gain-of-Function and Related Electro-Clinical Phenotypes

Antonella Riva ^1,^*, Loretta Ferrera ^1^, Chiara Reale ^2^, Ganna Balagura ^3^, Marcello Scala ^4^, Elisabetta Amadori ^4^, Maria Stella Vari ^5^, Francesca Madia ^6^, Elena Gennaro ^7^, Michele Iacomino ^6^, Irene Bagnasco ^8^, Gaetano Terrone ^9^, Emilia Ricci ^10^, Chiara Vannicola ^10^, Vincenzo Salpietro ^4^, Elena Gardella ^2^, Federico Zara ^1^ and Pasquale Striano ^4^

^1^ Unit of Medical Genetics, Department of Neurosciences, Rehabilitation, IRCCS Istituto Giannina Gaslini, Ophthalmology, Genetics, Maternal and Child Health, University of Genoa, Genoa, Italy
^2^ Danish Epilepsy Centre, Department of Epilepsy Genetics and Personalized Medicine, Dianalund, Denmark
^3^ Center for Neurogenomics and Cognitive Research (CNCR), Department of Functional Genomics, Vrije Universiteit (VU), de Boelelaan, 1081 HV Amsterdam, Amsterdam, The Netherlands
^4^ Paediatric Neurology and Muscular Disease Unit, Department of Neurosciences, IRCCS Istituto Giannina Gaslini, Rehabilitation, Ophthalmology, Genetics, Maternal and Child Health, University of Genoa, Genoa, Italy
^5^ Paediatric Neurology and Muscular Disease Unit, IRCCS Istituto Giannina Gaslini, Genoa, Italy
^6^ Unit of Medical Genetics, IRCCS Istituto Giannina Gaslini, Genoa, Italy
^7^ U.O.C Laboratorio di Genetica Umana, IRCCS Istituto Giannina Gaslini, Genoa, Italy
^8^ Division of Child Neuropsychiatry, Ospedale Martini, Turin, Italy
^9^ Section of Paediatrics—Child Neurology Unit, Department of Translational Medical Sciences, Università Federico II, Naples, Italy
^10^ Epilepsy Center, Health Sciences Department, Università degli Studi di Milano, Ospedale San Paolo, Milan, Italy
* Correspondence: riva.anto94@gmail.com

Purpose: Pathogenic variants in the *KCNB1* gene encoding the voltage-gated K+ channel (Kv) a-subunit are associated with a spectrum of phenotypes ranging from severe developmental and epileptic encephalopathies (DEE) to mild intellectual disability without epilepsy. Kv2.1 exerts an electrical role in neurons and mutations may be associated with loss of the channel voltage-dependence or loss of the ion currents conductance. We evaluated the functional effect of different pathogenic variants in *KCNB1* and correlated the results with the electro-clinical phenotype of patients.

Method: *KCNB1* pathogenic variants were identified through next-generation sequencing (NGS). Kv2.1 mutants were expressed in HEK293 cells and membrane currents were evaluated by patch-clamp technique. Cells were stimulated with constant pulse-potentials ranging from −80 to +120 mV, D = 20 mV (*n* ≥ 4 experiments for each mutation). Patients were deep-phenotyped through clinical charts collected from referring clinicians.

Results: We identified five *KCNB1* pathogenicvariants, including p.T210M, p.T804A, p.R293C, p.A406V, and p.F416L (four de novo, p.T804A inherited from the affected mother). In p.T804A mutant, Kv2.1 showed to stay open at >+50 mV potentials, whereas the Kv2.1 p.R293C mutant showed a different voltage-dependence and a modified reversal potential as compared to the wild-type channel (*p* = 9.35 × 10^−6^). Both variants were associated with a milder phenotype (focal epilepsy with mild intellectual disability) in two and eight patients, respectively. The remaining Kv2.1 mutants showed loss of the ion current conduction. These variants were found in 48 patients with severe phenotypes (DEE).

Conclusions: *KCNB1* mutations may impact patients’ phenotypes depending on the functional effect on the channel; milder phenotypes are more often associated with gain-of-function mutations, whereas severe phenotypes are associated with loss-of-function mutations.

## Septal Cholinergic Input to CA2 Hippocampal Region Controls Social Memory via Nicotinic Receptor-Mediated Disinhibition

Domenico Pimpinella ^1,^*, Valentina Mastrorilli ^2^, Corinna Giorgi ^3^, Silke Coemans ^1^, Hannah Ostermann ^4^, Elke C Fuchs ^4^, Hannah Monyer ^4^, Andrea Mele ^2^, Enrico Cherubini ^1^ and Marilena Griguoli ^5^

^1^ European Brain Research Institute, Neurophysiology, Roma, Italy
^2^ Center for Research in Neurobiology “D. Bovet,” Department of Biology and Biotechnology “C. Darwin,” Sapienza University of Rome, Roma, Italy
^3^ Institute of Molecular Biology and Pathology of the National Council of Research (IBPM-CNR), Roma, Italy
^4^ German Cancer Research Center (DKFZ), Department of Clinical Neurobiology, Medical Faculty of Heidelberg University, Heidelberg, Germany
^5^ European Brain Research Institute/Institute of Neuroscience of the National Research Council (IN-CNR), Pisa, Italy
* Correspondence: d.pimpinella@ebri.it

Despite its well-established neuromodulatory effect on attention, memory, and learning, the role of acetylcholine (ACh) in social memory, meaning the capacity of an animal to discriminate between a stranger and a familiar one, is virtually unknown. Medial Septum/Diagonal Band of Broca (MSDB) nuclei contain cholinergic neurons projecting to different brain areas, including the hippocampal CA2 region, which is known to be involved in social memory encoding. Here, different strategies have been used to control the activity of cholinergic neurons in the MSDB to evaluate its effect on social memory and on CA2 circuit functionality. Hence, AAVs carrying floxed tetanus toxin (TeNT); inhibitory and excitatory DREADD hM4 and hM3, respectively; or channelrhodopsin (ChR2) were stereotactically delivered in the MSDB of ChAT-Cre mice. After four weeks, behavioral (three-chamber test) and electrophysiological experiments were performed. ChAT-Cre mice expressing Tetanus toxin (TeNT) showed an impairment in the novelty discrimination task, an indicator of social memory. This effect was mimicked by both i.p. and hippocampal administration of CNO activating the inhibitory hM4 and by application of selective antagonist of nicotinic AChRs. Ex-vivo recordings from hippocampal slices, provided insight into the underlying mechanism, as activation of nAChRs by nicotine or CNO binding to hM3 increased the excitatory drive to CA2 principal cells via disinhibition. In line with this observation, optogenetic activation of cholinergic neurons in MSDB enhanced the firing of CA2 principal cells in vivo. In conclusion, our results point to nAChRs as essential players in social discrimination by controlling inhibition in the CA2 region.

## Chitosan Microstructures with Different Asymmetry for Peripheral Nerve Regeneration

Luca Scaccini ^1,^*, Roberta Mezzena ^1^, Mariacristina Gagliardi ^1^, Marco Cecchini ^1^, Ilaria Tonazzini ^1^, Alessia De Masi ^1^ and Giovanna Gambarotta ^2^

^1^ NEST, Scuola Normale Superiore, Pisa, Italy
^2^ Neuroscience Institute Cavalieri 9 Ottolenghi (NICO), University of Torino, Torino, Italy
* Correspondence: luca.scaccini@sns.it

Peripheral nerve injuries (PNIs) are a severe condition in which a nerve is damaged, affecting more than 300,000 people in Europe every year. Nowadays, there are still no efficient therapeutic treatments for PNIs. Artificial scaffolds can offer new opportunities for nerve regeneration applications, and in this framework, chitosan, an FDA-approved biomaterial, is emerging as promising. In this work, we propose a simple and effective method for the production of micro-structured chitosan films by solvent casting, with high fidelity in micro-pattern reproducibility. We developed three types of chitosan directional micro-grooved patterns, presenting different levels of symmetricity, for application in nerve regenerative medicine, including gratings (GR), isosceles triangles (ISO), and scalene triangles (SCA). We tested our directional topographies in vitro with the RT4D6P2T-GFP glial Schwann cell line. SCA, the most asymmetric topography, although leading to a less efficient cell polarization, promoted higher cell proliferation and a faster cell migration, both individually and collectively, with higher directional persistence of motion. In conclusion, the use of micro-structured asymmetrical directional topographies, produced on chitosan scaffolds, may be exploited to enhance the nerve regeneration process. Moreover, these results provide important information on the use of specific topographical features for other neural tissue engineering applications and for the fabrication of novel, bio-compatible neural scaffolds.

## Vulnerability and Resilience to Stress-Induced Anhedonic Phenotype Are Associated to a Different Modulation of Oxidative Balance

Vittoria Spero ^1,^*, Maria Serena Paladini ^1^, Eleonora Buscemi ^1^, Mariusz Papp ^2^ and Raffaella Molteni ^1^

^1^ Department of Medical Biotechnology and Translational Medicine, University of Milan, Milan, Italy
^2^ Polish Academy of Sciences, Institute of Pharmacology, Krakow, Poland
* Correspondence: vittoria.spero@unimi.it

Stress is an important environmental risk factor for the development or exacerbation of major depression, which affects around 10% of the world population. However, the impact of stress is highly variable, with the majority of the subjects exposed to adverse situations being resilient, able to positively cope with it, and a smaller percentage being susceptible, developing psychopathologies, and needing drug treatment. Although the molecular mechanisms underlying resilience and vulnerability are still elusive, their characterization is crucial to identify systems that might be the target of more efficient therapeutic strategies, able to correct the alterations observed in a vulnerable subject but also to favour the resilient response. To this aim, we evaluated, at preclinical level, the involvement of oxidative balance mediators, known to be altered in psychiatric disorders, in the differential response to the chronic mild stress (CMS) model of depression. Adult male rats were exposed to two weeks of CMS paradigm, and the sucrose intake test was used to assess the insurgence of an anhedonic phenotype. The animals were then divided in vulnerable, those developing the anhedonic phenotype, and the resilient that did not. A total of 24 h after the test, we performed the molecular analyses to evaluate the gene and protein expression of oxidative balance mediators. Our study shows that chronic stress has a significant impact on the balance between pro- and antioxidant factors in different cerebral areas implicated in psychopathologies. Interestingly, resilient animals showed a marked antioxidant response characterized by the Nrf2 pathway activation in the prefrontal cortex. Conversely, in vulnerable animals, we observed a region-specific increase in oxidative stress. Our results suggest the Nrf2 antioxidant pathway as a possible target to consider in developing new pharmacological therapies for stress-related disorders, as its activation could favour a positive behavioural and molecular response to stress.

## Assessment of Behavioural Outcomes after Stroke in a Mouse Model

Livia Vignozzi *, Stefano Varani and Matteo Caleo

Department of Biomedical Sciences, Università degli Studi di Padova, Padova, Italy
* Correspondence: livia.vignozzi@phd.unipd.it

Cerebral stroke is one of the major causes of disability worldwide. Spontaneous recovery of neurological function often occurs due to plastic rearrangements of perilesional spared tissue. However, recovery is highly variable in both individual patients and experimental animals and appears to depend on many factors (lesion location and volume, topology of structural and functional connectivity damage, etc.). Therefore, the need is great for a deeper understanding of the cellular plasticity mechanisms underlying neurological recovery as well as the identification of novel biomarkers that could represent predictive signals of recovery. In this scenario, a combination of multiple biomarkers able to document the processes of post-stroke plasticity and predict the extent of spontaneous recovery is needed to unravel the factors important to the recovery process and tailor specific treatments for individual patients. The aim of this project was to study the longitudinal evolution of both electrophysiological and behavioural parameters after a stroke in a mouse model. We used a Middle Cerebral Artery occlusion (MCAo) model to better represent human stroke characteristics and variability. Functional measures were taken from a peri-infarct zone, i.e., the forelimb primary motor cortex (caudal forelimb area, CFA). Electro-physiological measures were carried out in Thy1-ChR2 transgenic mice that express the light-gated cationic channel ChR2 mainly in layer V corticospinal neurons. We started with the creation of a behavioural protocol comprising Rotarod rotating test, Gridwalk, and Grip strength test to assess the degree of forelimb motor impairment. For the electrophysiological measures of neuronal plasticity, recordings of local field potentials (LFP) are carried out from the perilesional CFA following optogenetic stimulation delivered to either the contralateral CFA or the ipsilateral RFA (rostral forelimb area, equivalent to the premotor cortex).

## Data Availability

The data that support the findings of this abstract collection are available from the corresponding author, upon reasonable request.

